# cfDiffusion: diffusion-based efficient generation of high quality scRNA-seq data with classifier-free guidance

**DOI:** 10.1093/bib/bbaf071

**Published:** 2025-02-23

**Authors:** Tianjiao Zhang, Zhongqian Zhao, Jixiang Ren, Ziheng Zhang, Hongfei Zhang, Guohua Wang

**Affiliations:** College of Computer and Control Engineering, Northeast Forestry University, No. 26, Hexing Road, Xiangfang District, Harbin 150040, China; College of Computer and Control Engineering, Northeast Forestry University, No. 26, Hexing Road, Xiangfang District, Harbin 150040, China; College of Computer and Control Engineering, Northeast Forestry University, No. 26, Hexing Road, Xiangfang District, Harbin 150040, China; College of Computer and Control Engineering, Northeast Forestry University, No. 26, Hexing Road, Xiangfang District, Harbin 150040, China; College of Computer and Control Engineering, Northeast Forestry University, No. 26, Hexing Road, Xiangfang District, Harbin 150040, China; College of Computer and Control Engineering, Northeast Forestry University, No. 26, Hexing Road, Xiangfang District, Harbin 150040, China; Faculty of Computing, Harbin Institute of Technology, No. 92 Xidazhi Street, Nangang District, Harbin 150001, China

**Keywords:** scRNA-seq, data simulation, diffusion model, autoencoder

## Abstract

Single-cell RNA sequencing (scRNA-seq) technology provides a powerful means to measure gene expression at the individual cell level, thereby uncovering the intricate cellular heterogeneity that underlies various biological processes, including embryonic development, tumor metastasis, and microbial reproduction. However, the variable amounts of data generated across different cell types within tissues can compromise the accuracy of downstream analyses. Traditional approaches for generating scRNA-seq simulation data often rely on predefined data distributions, which can negatively impact the quality of the simulated data. Furthermore, these methods typically focus on simulating single-attribute cells, necessitating substantial additional data for the simulation of multi-attribute cells, which can lead to increased training times. To address these limitations, we propose cfDiffusion, a novel method grounded in diffusion models that incorporates Classifier-Free Guidance and a high-level feature caching mechanism. By leveraging Classifier-Free Guidance, cfDiffusion significantly reduces the training costs associated with model development compared to traditional Classifier Guidance methods. The integration of a caching mechanism further enhances efficiency by shortening inference times. While the inference duration of cfDiffusion remains longer than that of scDiffusion, it exhibits superior expressiveness and efficiency in generating multi-attribute single-cell data. Evaluated across datasets from multiple sequencing platforms, cfDiffusion consistently outperforms state-of-the-art models across various performance metrics. Additionally, cfDiffusion enables the simulation of single-cell data along a pseudo-time scale, facilitating advanced analyses such as tracking cell differentiation, investigating intercellular communication, and elucidating cellular heterogeneity.

## Introduction

Single-cell RNA sequencing (scRNA-seq) has revolutionized traditional gene expression analysis, enabling high-throughput, comprehensive characterization of individual cell transcriptomes. Research utilizing scRNA-seq data plays a crucial role in various aspects. scRNA-seq aids in identifying and characterizing different cell types within heterogeneous cell populations [1, 2], recognizing cancer cell heterogeneity [3] and developing personalized therapies [4], understanding the complexity of immune cell populations and their responses to stimuli [5], dissecting stem cell differentiation and regulation, discovering stem cell markers, and deeply comprehending the regulatory networks controlling gene expression [6].

In downstream analyses of scRNA-seq data, sufficient high-quality scRNA-seq data aids researchers in drawing relatively accurate conclusions. However, factors such as the high cost of sequencing, low measurement accuracy of rare cell types, and data privacy concerns can affect the quantity and quality of sequencing data, potentially impacting the accuracy of subsequent analyses. To address this issue, researchers can conduct multiple experiments using different sequencing technologies to collect more samples. This approach not only increases experimental costs but also introduces batch effects when integrating data from different sequencing platforms, which can compromise data quality. Additionally, due to privacy protection and other reasons, it is often challenging for researchers to augment existing data.

In recent years, an increasing number of researchers have proposed various computational methods to generate high-quality scRNA-seq data. These methods include deep learning generative algorithms such as LSH-GAN [7], scGAN [8], sciGANs [9], and scDiffusion [10], as well as statistical frameworks like Splatter [11], which simulate single-cell samples of specific cell types or subpopulations. These approaches provide different insights into the challenge of obtaining high-dimensional, high-quality scRNA-seq data. However, most statistical methods assume that the distribution of single-cell data follows Gaussian, ZINB [12], or other distributions. This assumption lacks robustness and may overlook potential biological significance, as the distribution of single-cell data is usually unknown and assumed distributions may fail to accurately simulate the data. This could lead to biased results, especially when the assumed distribution deviates significantly from the true distribution. Deep generative models can learn the biological patterns of single-cell gene expression and build corresponding models, making it possible to generate realistic scRNA-seq data. VQVAE [13] enables efficient inference, making it suitable for large-scale data applications. The discrete latent space and vector quantization process help prevent overfitting, resulting in more generalizable models. However, VQVAE relies on a fixed set of discrete code vectors, which may limit its ability to capture complex distributions. Consequently, models like scVI [14], which use VQVAE as the network framework, are not well-suited for generating diverse single-cell gene expression data. Furthermore, scVI assumes that the data follows a Zero-Inflated Negative Binomial (ZINB) distribution; however, the actual distribution of scRNA-seq data is highly complex. Thus, imposing such distributional assumptions on the data is not reasonable. Moreover, since our objective is to enhance the raw data, this approach may introduce additional noise, which could be detrimental to downstream analyses. GANs [15] are particularly well-suited for tasks that require the generation of highly realistic and diverse samples, such as image synthesis, data augmentation, or style transfer. For example, scGAN employs deep learning models to learn the nonlinear relationships between genes from different cell samples, then generates realistic scRNA-seq data based on the learned information, achieving impressive results across many evaluation metrics. The adversarial training process involves iteratively updating both networks: the generator improves its ability to produce realistic samples to fool the discriminator, while the discriminator enhances its capability to accurately classify real and fake samples. This competition drives both networks towards convergence, ideally resulting in a generator that produces data indistinguishable from real samples when evaluated by the discriminator. Although GANs can model complex distributions, their training process is often unstable and may suffer from mode collapse, where the generator produces limited diverse data and fails to capture the full diversity of the data distribution. To mitigate this issue, careful design of the GAN network structure, hyperparameter selection, and loss function is required.

The Diffusion model [16] is capable of flexibly generating realistic images, audio, and text, and it is more expressive compared to GANs. It has been applied to large models and various applications such as Stable Diffusion [17], Grad-tts [18], Diffwave [19]. Diffusion is more stable during training and can learn complex data distributions. Single-cell data is not only high-dimensional but also exhibits different distributions across various cell types. Using Diffusion as a framework allows for the autonomous learning of single-cell gene expression data distributions without presupposing any specific probability distribution. This capability effectively generates single-cell data while preserving biological specificity. In gene expression matrices, the dependency between adjacent genes is relatively weak, whereas in images, adjacent pixels have strong dependencies. Therefore, replacing CNNs with MLPs [20] can yield better generative results. Although Diffusion shows great potential for generating higher quality images compared to GANs, its slow sampling speed hinders its widespread practical application. Strategies to accelerate model inference primarily focus on model pruning [21], model distillation [22], and model quantization [23]. Model pruning increases efficiency by removing redundant or insignificant weights to reduce model size. However, The search for the optimal pruning rate is difficult because there are risks of over-pruning or under-pruning. Model distillation involves transferring knowledge from a large, complex teacher model to a smaller, simpler student model. The performance of the student model depends heavily on the quality of the teacher model, and training student models through distillation requires high computational costs. Model quantization reduces the precision of model weights and activations to decrease memory usage. However, quantization can lead to underflow or overflow, making it inappropriate for models with very large or very small weights. To overcome these challenges, this study introduces a mechanism for caching high-level features. This ensures the generation quality of single-cell data and operational simplicity while speeding up model inference.

This study faces two major challenges: ensuring the quality of generated data while accelerating the inference process of Diffusion models without introducing additional training costs, and generating high-quality single-cell gene expression data of specific types without classifier gradient guidance. Ma, et al. [24] revealed that the high-level features between consecutive time steps in the reverse process possess similarities, and proposed to save and reuse the high-level features of a certain time step in subsequent time steps under the U-Net [25] architecture, with periodic updates of high-level features. This strategy does not incur any additional training costs and significantly improves the efficiency and speed of Diffusion inference. Furthermore, this model adopts the Classifier-free [26] principle to guide the Diffusion model to generate high-quality, high-dimensional single-cell gene expression data of specific cell types under multiple or single conditions.

## Materials and methods

### cfDiffusion framework

**Figure 1:**
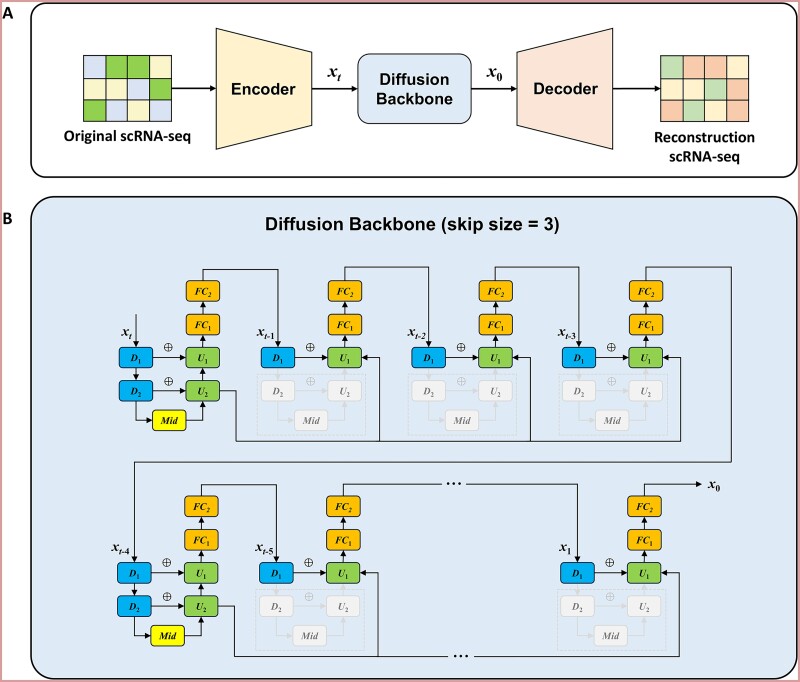
The framework of the cfDiffusion model for generating single-cell gene expression data. (a) The cfDiffusion model framework consists of an encoder, a diffusion model, and a decoder. The encoder extracts important features from gene expression data, the diffusion model progressively adds noise and denoises these features, and the decoder reconstructs the final gene expression data. (b) the diffusion backbone network architecture, designed based on the UNet model, comprises modules *D*_1_,*D*_2_,*U*_1_,*U*_2_,*FC*_1_,*FC*_2_,*mid*, all of which employ MLP (multilayer perceptron). *x_t_* represents the feature information at a specific time step. The model accelerates the generation of single-cell gene expression data during inference when the number of skip connection is 3 and the specified caching module is *U*_2_. First, the feature information at time step t is cached from module *U*_2_. In the subsequent time steps *t-1,t-2,t-3*, the cached feature information is input into module *U_1_*, which processes the feature information and then fuses it with the feature information from module *D_1_*. Throughout the feature processing process, only modules *D_1_, U_1_, FC_1_, FC_2_* are involved, without passing through the gray modules (*D_2_,mid,U_2_*). The operation process of time step *t-4* is the same as that of time step *t*, achieving an update of the cached information.

The cfDiffusion framework inspired by scDiffusion, consists of two components: an autoencoder (AE) and a Diffusion model, as shown in Fig. 1a. Due to the high-dimensional and sparse nature of gene expression data, we employ an AE to denoise and reduce the dimensionality of the original data. The AE comprises an encoder and a decoder. The encoder maps the input data to a low-dimensional latent representation, while the decoder reconstructs the original data from the latent features. By optimizing the reconstruction loss, the AE can effectively extract the low-dimensional key features that preserve the essential information in the original data. The trained AE encoder is then used to extract features and reduce the dimensionality of the input data. The resulting latent features not only have a lower dimensionality but also encapsulate the critical semantic information of the original data.

**Algorithm 1:** Improved U-net implement, given ${x}_t,t,y.$

Input: class label $y$, embedding ${x}_t$, time $t$, Chunk size $n$ Output: ${\boldsymbol{\epsilon}}_{\boldsymbol{\theta}}\left({x}_t,t,y\right)$ Stack stack$\leftarrow \left\{\phi \right\}$ //Initialize an empty stack **for all** $i$ from $1$ to $n$ **do** ${x}_t\leftarrow{x}_t\bigodot y+t$ **if** ${Chunk}_i$ $\in$ Down sample Chunck: put ${x}_t$ in stack **else if** ${Chunk}_i$ $\in$ Up sample Chunk: take ${x}_s$ from the top of stack stack pop //Drop the top element ${x}_t\leftarrow{x}_t+{x}_s$
$\epsilon = FC\left({x}_t\right)$ **return** $\epsilon$

In the training of the diffusion model, the latent features extracted by the AE are used as input, along with the corresponding class label information. The diffusion model learns the transformation mapping from noise to the target distribution through an iterative process of adding and removing noise. During this process, the class label information is incorporated through a Classifier-Free mechanism, guiding the generation of the latent features to align more closely with the feature distribution of the target class. Finally, the trained diffusion model can transform random noise into new latent feature representations corresponding to a specified cell type, and the AE decoder can then reconstruct the corresponding gene expression data samples.

To accelerate the inference process, we introduce a caching mechanism for high-level features, as detailed in the Diffusion Inference Acceleration Section. In summary, the diffusion model is responsible for generating the latent features of the gene expression data, while the AE is responsible for reconstructing the original data from the latent features and extracting the latent features from the original data. The combination of the autoencoder and diffusion architecture enables more efficient training and inference, as well as reduced computational resource requirements. If only the diffusion model was used for data generation, it would have to handle high-dimensional and noisy gene expression data, making the data generation process more challenging and resource-intensive.

### Diffusion overview

Given a sample ${\mathbf{x}}_0\sim q\left(\mathbf{x}\right)$ from the true data distribution, we gradually add Gaussian noise $\epsilon$ over $T$ continuous time steps, with the size of each time step controlled by ${\left\{{\beta}_t\in \left(0,1\right)\right\}}_{t=1}^T$, as shown in Equation (1):


(1) \begin{equation*} q\left({\mathbf{x}}_t|{\mathbf{x}}_{t-1}\right)=\mathcal{N}\left({\mathbf{x}}_t;\sqrt{1-{\beta}_t}{\mathbf{x}}_{t-1},{\beta}_t\mathrm{I}\right)\kern1.25em q\left({\mathbf{x}}_{1:T}|{\mathbf{x}}_0\right)=\prod_{t=1}^Tq\left({\mathbf{x}}_t|{\mathbf{x}}_{t-1}\right) \end{equation*}

where $\mathrm{I}$ follows a standard normal distribution. When $T\to \infty$, ${\mathbf{x}}_T$ approximately follows a Gaussian distribution. We parameterize the Diffusion model as ${\epsilon}_{\theta}\left({\mathbf{x}}_t,t\right)$, and during training, the Diffusion model learns to progressively denoise ${\mathbf{x}}_t$ to ${\mathbf{x}}_0$, modeling the process ${p}_{\theta}\left({\mathbf{x}}_{t-1}|{\mathbf{x}}_t\right)$ as a diagonal Gaussian $\mathcal{N}\left({\mathbf{x}}_{t-1};{\mu}_{\theta}\left({\mathbf{x}}_t,t\right),{\Sigma}_{\theta}\left({\mathbf{x}}_t,t\right)\right)$, where we view ${\mu}_{\theta}\left({\mathbf{x}}_t,t\right)$ as ${\epsilon}_{\theta}\left({\mathbf{x}}_t,t\right)$, and ${\Sigma}_{\theta}\left({\mathbf{x}}_t,t\right)$ is fixed to a known constant or learned as a parameter using a neural network. The optimization objective is ${\left\Vert{\epsilon}_{\theta}\left({\mathbf{x}}_t,t\right)-\epsilon \right\Vert}^2$, i.e., the mean squared error between the predicted noise and the true noise. In the inference stage, we cannot directly denoise the random Gaussian noise ${\mathbf{x}}_t^{\prime }$ to obtain ${\mathbf{x}}_0^{\prime }$, but rather progressively denoise from $t=1$ to obtain the final sample, according to the process ${p}_{\theta}\left({\mathbf{x}}_{t-1}^{\prime }|{\mathbf{x}}_t^{\prime}\right)$:


(2) \begin{equation*} {\mathbf{x}}_{t-1}^{\prime}\sim{p}_{\theta}\left({\mathbf{x}}_{t-1}^{\prime }|{\mathbf{x}}_t^{\prime}\right)=\mathcal{N}\left({\mathbf{x}}_{t-1}^{\prime };\frac{1}{\sqrt{\alpha_t}}\left({\mathbf{x}}_t^{\prime }-\frac{1-{\alpha}_t}{\sqrt{1-{\overline{\alpha}}_t}}{\epsilon}_{\theta}\left({\mathbf{x}}_t^{\prime },t\right)\right),{\beta}_t\mathrm{I}\right) \end{equation*}

Where ${\overline{\alpha}}_t=\prod_{i=1}^t{\alpha}_t$，${\alpha}_t=1-{\beta}_t$.

### AE training

The gene expression matrix ${X}_{n\times m}$ is normalized to obtain ${S}_{ori}$, which is used as the input to the AE. Specifically, each cell’s total count is scaled to 10 000, and then a small offset of 1 is added, followed by taking the logarithm.


(3) \begin{equation*} {S}_{ori}=\ln \left(\frac{\mathrm{X}}{10^4}+1\right) \end{equation*}

This normalization step not only helps reduce the differences in sequencing depths between cells and the impact of extreme values on analysis but also stabilizes the data's variance, transforming the original data distribution into an approximate normal distribution. This is in line with the Gaussian distribution used in the diffusion process, making it easier for the Diffusion model to learn the reverse process. The AE's Encoder consists of two MLP layers, which processes ${S}_{ori}$ to obtain a 128-dimensional embedding ${x}_0$:


(4) \begin{equation*} {x}_0= Encoder\left({S}_{ori}\right) \end{equation*}

The AE's Decoder consists of three MLP layers, which takes ${x}_0$as input and outputs a reconstructed gene expression matrix ${S}_{rec}$ with the same dimensions as ${S}_{ori}$:


(5) \begin{equation*} {S}_{rec}= Decoder\left({x}_0\right) \end{equation*}

The optimization goal is to reconstruct ${S}_{rec}$ as closely as possible to ${S}_{ori}$:


(6) \begin{equation*} Loss={\left\Vert{S}_{rec}-{S}_{ori}\right\Vert}_F^2 \end{equation*}

### Classifier-free diffusion training

In the forward process of Diffusion, noise is added to the embedding ${x}_0$ at a series of random time steps. According to formula (1), the embedding ${x}_t$ at the t-th time step can be obtained:


(7) \begin{equation*} {x}_t=\sqrt{\alpha_t}{x}_{t-1}+\sqrt{1-{\alpha}_t}{\boldsymbol{\epsilon}}_{t-1}=\sqrt{{\overline{\alpha}}_t}{x}_0+\sqrt{1-{\overline{\alpha}}_t}\boldsymbol{\epsilon} \end{equation*}

where ${\boldsymbol{\epsilon}}_{t-1},{\boldsymbol{\epsilon}}_{t-2},{\boldsymbol{\epsilon}}_{t-3},\dots \sim \mathcal{N}\left(\mathbf{0},\mathbf{I}\right)$, and $\boldsymbol{\epsilon}$ is a fusion of $t-1$ Gaussian distributions. When $t$ is large enough, ${x}_t$ approximately follows a Gaussian distribution. Due to the high sparsity and high dimensionality of the gene expression matrix, directly using U-Net’s convolutional operations cannot effectively capture the relationships between two genes that are far apart in the vector, or the captured relationships are weak. MLP can resolve this issue, so using fully connected networks instead of convolutional layers can improve the performance of generating single-cell data using Diffusion. An improved U-Net is shown in Fig. 1b. Time information $t$ and label information $y$ are incorporated into each chunk, and the first chunk is used as an example, as shown in formula (8):


(8) \begin{equation*} {x}_t={x}_t\bigodot y+t \end{equation*}

where $\bigodot$ is the matrix dot product operation. Each chunk updates ${x}_t$. In the improved U-Net, the number of chunks is set to $n$, and $n$ is an odd number. The information from the first $\frac{n-1}{2}$ chunks is passed through skip connections, directly connecting the low-level information to the last $\frac{n-1}{2}$ chunks, as shown in formula (9):


(9) \begin{equation*} {U}_{i+1}={U}_i\left(\cdot \right)+{D}_i\left(\cdot \right) \end{equation*}

where $i=1,2\dots \frac{n-1}{2}$, ${U}_i$ is the last $\frac{n-1}{2}$ chunks, ${D}_i$ is the first $\frac{n-1}{2}$ chunks, and the $\frac{n+1}{2}$-th chunk is equivalent to the intermediate layer of the U-Net network. The entire process of ${x}_t$, $t$, and $y$ passing through the improved U-Net is shown in pseudo-code Algorithm 1.

Training the Diffusion model to learn the reverse process of denoising from ${x}_t$ to ${x}_{t-1}$, which can be represented as $p\left({x}_{t-1}|{x}_t\right)$. The Diffusion model is parameterized as ${\boldsymbol{\epsilon}}_{\boldsymbol{\theta}}$, and label information $y$ is incorporated during training. Assuming ${\boldsymbol{\epsilon}}_{\boldsymbol{\theta}}\left({x}_t,t,y\right)$ follows a standard normal distribution, according to formula (7), we can obtain:


(10) \begin{equation*} {x}_t=\sqrt{{\overline{\alpha}}_t}{x}_0+\sqrt{1-{\overline{\alpha}}_t}{\boldsymbol{\epsilon}}_{\boldsymbol{\theta}}\left({x}_t,t,y\right) \end{equation*}

In fact, the noise ${\boldsymbol{\epsilon}}_{\boldsymbol{\theta}}$ learned by Diffusion is approximately normally distributed. Using the Tweedie Estimator, we can further derive formula (11):


(11) \begin{equation*} {x}_t=\sqrt{{\overline{\alpha}}_t}{x}_0-\left(1-{\overline{\alpha}}_t\right){\nabla}_{x_t}\log p\left({x}_t|y\right) \end{equation*}

Then, using the Bayes’ formula and taking the logarithm, we can obtain:


(12) \begin{align*} \log p\left({x}_t|y\right)&=\log p\left(y|{x}_t\right)+\log p\left({x}_t\right)-\log p(y)\nonumber \\{}{\nabla}_{x_t}\log p\left(y|{x}_t\right)&={\nabla}_{x_t}\log p\left({x}_t|y\right)-{\nabla}_{x_t}\log p\left({x}_t\right)+{\nabla}_{x_t}\log p(y) \nonumber \\{}&={\nabla}_{x_t}\log p\left({x}_t|y\right)-{\nabla}_{x_t}\log p\left({x}_t\right) \nonumber \\{}&=-\frac{1}{\sqrt{1-{\overline{\alpha}}_t}}{\boldsymbol{\epsilon}}_{\boldsymbol{\theta}}\left({x}_t,t,y\right)+\frac{1}{\sqrt{1-{\overline{\alpha}}_t}}{\epsilon}_{\theta}\left({x}_t,t,y=\varnothing \right) \end{align*}

Introducing a hyperparameter $k$, we can get:


(13) \begin{align*} {\hat{\boldsymbol{\epsilon}}}_{\boldsymbol{\theta}}\left({x}_t,t,y\right) &= {\boldsymbol{\epsilon}}_{\boldsymbol{\theta}}\left({x}_t,t,y\right)-k\sqrt{1-{\overline{\alpha}}_t}{\nabla}_{x_t}\log p\left(y|{x}_t\right) \nonumber \\{}&={\boldsymbol{\epsilon}}_{\boldsymbol{\theta}}\left({x}_t,t,y\right)-k\left({\boldsymbol{\epsilon}}_{\boldsymbol{\theta}}\left({x}_t,t,y=\varnothing \right)-{\boldsymbol{\epsilon}}_{\boldsymbol{\theta}}\left({x}_t,t,y\right)\right) \nonumber \\{}&=\left(1-k\right){\boldsymbol{\epsilon}}_{\boldsymbol{\theta}}\left({x}_t,t,y\right)-k{\boldsymbol{\epsilon}}_{\boldsymbol{\theta}}\left({x}_t,t,y=\varnothing \right) \end{align*}

The hyperparameter $k$ controls the trade-off between fidelity and diversity of the generated data, with a value range from 1 to 2.

### Diffusion inference acceleration

Not In the reverse diffusion process, the features ${x}_i$ and its neighboring features ${x}_{i-1}$, ${x}_{i-2}$, etc. are similar. In continuous time steps, we can skip redundant computations by jumping over certain U-Net layers. To better understand the entire accelerated inference process, we set the number of jumps to 3 (as shown in Fig. 1b). Each jump goes to the ${chunk}_5$, which is the upsampling layer ${U}_1$. The features on ${U}_i$ are called high-level features, and the features on ${D}_i$ are called low-level features. A certain high-level feature will be saved for jump calculation. As shown in Fig. 1b, we can observe that at the $T$-th time step, the feature on ${U}_1$ is saved as ${x}_{save}$, and ${x}_t$ goes through the entire U-Net and denoising to get the embedding ${x}_{t-1}$. At the $\left(T-1\right)$-th time step, ${x}_{t-1}$ does not go through ${D}_2$,$Mid$,${U}_1$ layers, but directly goes through ${D}_1$ and ${U}_2$, and calculates ${\epsilon}_{t-2}$ as shown in formula (14):


(14) \begin{equation*} {\epsilon}_{t-2}={FC}_2\left\{{FC}_1\left[{D}_1\left({x}_{t-1},t-1,y\right)+{U}_2\left({x}_{save},t-1,y\right)\right]\right\} \end{equation*}

Finally, we get ${x}_{t-2}$ by further denoising:


(15) \begin{equation*} {x}_{t-2}= Denoise\left({x}_{t-1},{\epsilon}_{t-2}\right) \end{equation*}

In the $\left(T-2\right)$-th and $\left(T-3\right)$-th time steps, we can get ${x}_{t-3}$, ${x}_{t-4}$ using the same jump calculation method, and so on. In the $\left(T-4\right)$-th time step, ${x}_{t-4}$ needs to go through the entire U-Net to update ${x}_{save}$, and then uses the same jump calculation method to get ${x}_{t-5}$, ${x}_{t-6}$, ${x}_{t-7}$. And so on, until we get ${x}_0$.

Now, we generalize all conditions. First, we define a sequence $\xi =\left\{{p}_1,{p}_2,\dots, {p}_m\right\}$ that updates ${x}_{save}$ at certain time steps, where ${p}_i\in \left[\mathrm{0,999}\right]$, $m\le 1000$. If the jump step is fixed, then $\left|{p}_i-{p}_{i+1}\right|=c$, where $c$ is a constant. If the jump step is dynamically changing, then $P\left(|{p}_i-{p}_{i+1}|=|{p}_{i+1}-{p}_{i+2}|\right)>0$, where $P\left(\cdot \right)$ represents the probability of an event. To specify the jump to an arbitrary ${U}_b$, where $b$ represents the index, we note that the U-Net structure is symmetric, meaning ${U}_b$ corresponds to chunk ${D}_b$. The update of ${x}_{save}$ and the jump process are illustrated in Equation (16):


(16) \begin{equation*} \left\{\begin{array}{@{}ll}{x}_{save}={U}_{b-1}^t\left(\cdot \right), & t\in \xi \\{}{U}_b^t\left({x}_{save},t,y\right)+{x}_{save}, & t\notin \xi \end{array}\right. \end{equation*}

where ${U}_{b-1}^t$ represents the high-level feature at time step $t$ with index $b-1$.

### Datasets and preprocessing

We summarize the datasets in Table 1. Each dataset is described in more detail in Supplementary Dataset.

**Table 1:** The scRNA-seq dataset used in this paper

Dataset name	Genes	Cells	Cell types	Zero ratio
Tabula Muris [27]	18,996	57,004	12	90.11%
PBMC68k [28]	17,789	68,579	11	96.94%
Waddington-OT [29]	19,423	82,920	15	83.61%
*Homo Sapiens* [10]	28,231	46,177	(5, 6)^*^	90.35%
Human_PF_Lung [10]	27,281	114,396	31	91.42%
muris_mam_spl_T_B [10]	14,652	11,330	(2, 2)^*^	94.47%
Alles [30]	14,850	4614	14	90.41%
Baron [31]	16,359	8569	14	88.46%
MarShall [32]	16,548	6022	16	87.77%
Mizrak [33]	48,529	42,374	17	97.40%

^a^(5, 6)^*^ indicates 6 cell types in 5 tissues and (2, 2)^*^ indicates 2 cell types in 2 tissues.

### Evaluation metrics

Note: To quantify the similarity between generated cells and real cells, we employed both numerical and non-numerical metrics. Numerical metrics used in this study include Pearson Correlation Coefficient (PCC), Maximum Mean Discrepancy (MMD), Local Inverse Simpson Index (ILISI), Spearman Correlation Coefficient (SCC), and Quantile-Quantile Plot (QQ-plot). Non-numerical metrics include Uniform Manifold Approximation and Projection (UMAP) visualization and random forest classification of single-cell real and fake data.

Spearman Correlation Coefficient: The SCC is used to measure the correlation between the gene expression levels of generated data ${Y}_{n\times m}$ and real data ${X}_{n\times m}$. The mean expression levels of each gene in both samples are calculated as:


(17) \begin{align*} {X}_{i\times 1}^{\prime}=& \frac{1}{m}\sum_{j=1}^m{X}_{ij}\nonumber \\{}\ {Y}_{i\times 1}^{\prime }=&\frac{1}{m}\sum_{j=1}^m{Y}_{ij} \end{align*}

where $n$ represents the number of cells, $m$ represents the number of genes. We calculate SCC as:


(18) \begin{equation*} \rho =1-\frac{6\sum_i{\left|R\left({X}_{i\times 1}^{\prime}\right)-R\left({Y}_{i\times 1}^{\prime}\right)\right|}^2}{n\left({n}^2-1\right)} \end{equation*}

where $R\left({X}_{i\times 1}^{\prime}\right)$ and $R\left({Y}_{i\times 1}^{\prime}\right)$ are the ranks of ${X}_{i\times 1}^{\prime }$ and ${Y}_{i\times 1}^{\prime }$, respectively. The SCC $\rho$ ranges from −1 to 1, where $\rho$ close to −1 indicates strong negative correlation, and $\rho$ close to 1 indicates strong positive correlation. In all experiments, the SCC was greater than 0.9, indicating a strong positive correlation between the average gene expression levels of generated data and real data.

Pearson Correlation Coefficient: The PCC evaluates the linear correlation between the average gene expression levels in generated data and real data. The calculation formula is:


(19) \begin{equation*} r=\frac{\sum_{i=1}^n\left({X}_{i\times 1}^{\prime }-{\overline{X}}^{\prime}\right)\left({Y}_{i\times 1}^{\prime }-{\overline{Y}}^{\prime}\right)}{\sqrt{\sum_{i=1}^n{\left({X}_{i\times 1}^{\prime }-{\overline{X}}^{\prime}\right)}^2\sum_{i=1}^n{\left({Y}_{i\times 1}^{\prime }-{\overline{Y}}^{\prime}\right)}^2}} \end{equation*}

where ${\overline{X}}^{\prime }=\frac{1}{n}\sum_{i=1}^n{X}_{i\times 1}^{\prime }$，${\overline{Y}}^{\prime }=\frac{1}{n}\sum_{i=1}^n{Y}_{i\times 1}^{\prime }$. The PCC $r$ close to 1 indicates that the gene expression patterns of generated single-cell data are similar to those of real single-cell data.

Maximum Mean Discrepancy: MMD is used to highlight the differences between the distributions of two samples. It exists a well-behaved function $f:x\mapsto \mathbb{R}\in \mathcal{F}$ that computes the maximum mean discrepancy between the two samples:


(20) \begin{align*} & MMD =\underset{f\in \mathcal{F}}{{max}}\kern0.1em \left|{\mathbb{E}}_{X_{pc}\sim P\left({X}_{pc}\right)}f\left({X}_{pc}\right)-{\mathbb{E}}_{Y_{pc}\sim Q\left({Y}_{pc}\right)}f\left({Y}_{pc}\right)\right| \nonumber \\ & \hat{MMD}=\underset{f\in \mathcal{F}}{{max}}\kern0.1em \frac{1}{n}\sum_{X_{pc}}\kern0.1em f\left({X}_{pc}\right)-\frac{1}{n}\sum_{Y_{pc}}\kern0.1em f\left({Y}_{pc}\right) \end{align*}

where ${X}_{pc}= PCA(X)$, ${Y}_{pc}= PCA(Y)$. We used principal component analysis to reduce the dimensionality of the real data and generated data, which helps alleviate the computational burden.

Local Inverse Simpson Index: The ILISI is a metric used to evaluate the diversity of single-cell datasets. It is based on the Simpson Index, which is a measure of diversity in ecology. In single-cell analysis, ILISI assesses the inverse probability of two cells originating from the same cell type. A higher ILISI value (> 0.5) indicates a more diverse dataset, suggesting high-quality data that are well-integrated with the real data. The calculation is implemented using the `scib.me.ilisi_graph` function in Python.

Quantile-Quantile Plot: The QQ-plot is a widely applicable graphical method for comparing the distributions of two samples by plotting their quantiles against each other. If the generated data and real data are similar, the QQ-plot tends to fall along the $ y=x $ line.

Uniform Manifold Approximation and Projection: UMAP is a nonlinear dimensionality reduction and visualization algorithm. It constructs a graph of neighboring relationships between data points and utilizes the graph’s topological structure to approximate and optimize the manifold, enabling the visualization of high-dimensional data. By reducing the data to 2 dimensions using UMAP, we can observe the distribution of the generated data and real data.

Random Forest: To evaluate the similarity between real and generated data, we label the real data as true and the generated data as fake. We then mix the two samples randomly to create a larger dataset, which is divided into 5 folds. Four folds are used to train a random forest model, and the remaining fold is used for model inference. The evaluation metric is Accuracy:


(21) \begin{equation*} Accuracy=\frac{TP+ TN}{TP+ TN+ FP+ FN} \end{equation*}

A higher Accuracy value approaching 0.5 indicates that the random forest model struggles to distinguish between real and generated data, reflecting the effectiveness of the diffusion model in learning the data's features.

K-Nearest Neighbors: In addition to utilizing the random forest method to assess the quality of simulated data, we also employed the K-Nearest Neighbors (KNN) algorithm for evaluation. In this process, we set the ratio of training data to testing data at 7:3. The closer the prediction accuracy of the KNN algorithm is to 0.5, the higher the quality of the simulated data. Furthermore, we calculated the Receiver Operating Characteristic (ROC) curve and its corresponding Area Under the Curve (AUC) score to provide a more comprehensive assessment of the quality of simulated data.

The Adjusted Rand Index (ARI) is a measure used to evaluate the consistency of clustering results by comparing the similarity between the clustering outcome and true labels. The value of ARI ranges from −1 to 1, where higher values indicate greater agreement between the clustering result and the true labels. The adjustment mechanism accounts for the total number of sample pairs, thus mitigating accidental agreements in random clustering.


(22) \begin{equation*} ARI=\frac{RI-E(RI)}{\max (RI)-E(RI)} \end{equation*}

Normalized Mutual Information (NMI) measures the shared information between clustering results and true labels. The value of NMI ranges from 0 to 1, where 0 signifies no mutual information and 1 signifies perfect alignment between clustering results and true labels. NMI integrates the concepts of mutual information and entropy to effectively evaluate the performance of clustering.


(23) \begin{equation*} NMI\left(U,V\right)=\frac{2I\left(U;V\right)}{H(U)+H(V)} \end{equation*}

$I\left(U;V\right)$ is the mutual information between clustering result$U$ and true labels $V$. $H(U)$ and $H(V)$ are the entropy of the clustering result and the true labels, respectively.

In an ideal scenario, we anticipate that a decrease in MMD and Wasserstein distances, coupled with an increase in ILISI, would result in the visualization of simulated data distributions being more aligned with real data in the UMAP plot. This relationship is also expected to correlate with higher PCC and SCC values.

Our experimental results generally corroborate this expectation. For instance, in the datasets muris_T_B, Baron_Human, Marshall, sapiens, and Human_PF_Lung, cfDiffusion exhibited the smallest MMD and Wasserstein distances alongside the highest ILISI value, indicating that the distribution of cfDiffusion's simulated data is the closest to that of the real data in the UMAP visualizations. In some cases, such as in the WOT and Alles datasets, cfDiffusion performed optimally in terms of ILISI and MMD, while its Wasserstein distance was only approximately 0.01 lower than that of the top-ranking method. Notably, across all datasets, both SCC and PCC values for cfDiffusion consistently exceeded 0.9.

## Results

### Accelerating inference with skipping

**Figure 2:**
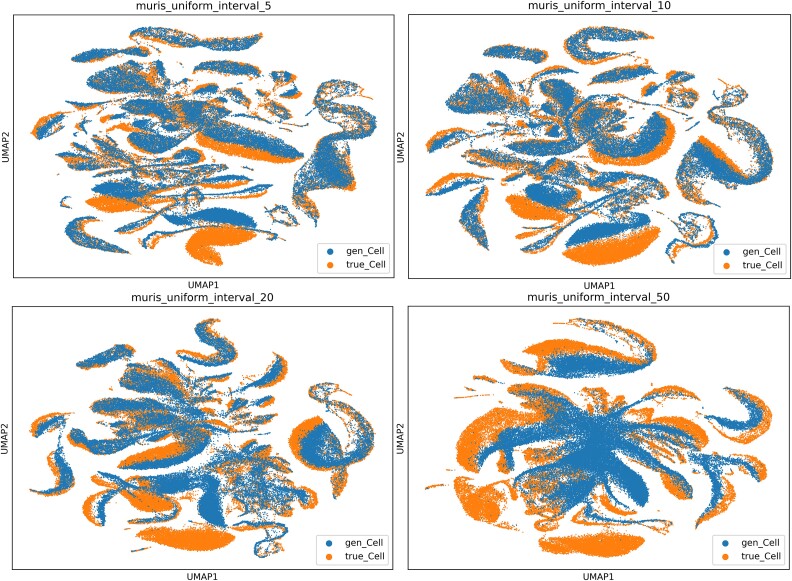
UMAP visualization of real data and generated data in Muris datasets on a 2D space, showing changes in generated data quality when the model skipping step size is 5, 10, 20, and 50.

During the inference stage of the model, using a classifier-free guided generation approach to generate specific types of single-cell gene expression data can be computationally expensive. To accelerate the inference speed, we adopted a skipping strategy. However, if the skipping interval is too small, the comparison of experimental results may not be evident. Therefore, we set the skipping interval to start from 5. As shown in Fig. 2, Supplementary Table 1 and Supplementary Fig. 5, the quality of generated single-cell gene expression data changes as the skipping interval increases. The most prominent trend is that the time cost decreases gradually, as shown in Supplementary Table 2. According to Supplemental Table 1, when the skipping interval increases from 5 to 20, the quality of generated single-cell expression data decreases slightly, indicating that the features between adjacent time steps are similar and there is some redundancy. When the skipping interval is 50, the quality of generated single-cell expression data degrades significantly, suggesting that some important features are deleted. Although the data quality decreases within the skipping interval of 5–20 steps, the overall performance of cfDiffusion is not inferior to scDiffusion from the perspective of evaluation metrics. For example, in the experiment on the WOT dataset, the random forest model finds it harder to distinguish cfDiffusion-generated data, and the increased ILISI index also indicates better integration of cfDiffusion-generated data with the original data.

We also explored the correlation of gene expression between generated cells and real cells in the pbmc68k and Muris datasets, visualized by QQ-plots (Supplemental Figs. 1, 2). In the Muris dataset, we selected transcription factors with the highest average Gini index (Cnbp, Hnrnpk, Klf13, Ybx1) referenced scDiffusion. In the PBMC68k dataset, we selected two marker genes (CD3D, NKG7). We z-scored the entire gene expression matrix and plotted the curves. The closer the curves of different colors are to the middle dashed line, the more similar the gene expression distributions are between generated cells and real cells.

### Comparison of the quality of scRNA-seq data generation on the same cell type dataset from different tissues

Traditional multi-attribute data generation techniques often rely on supervised classifiers to condition the generative model based on known attributes. This reliance may require extensive labeled datasets for effective training. The training data consists of original data, data generated by adding noise to the original data at each time step, and corresponding data labels. Each attribute of the data corresponds to a classifier, and for data with multiple attributes, a substantial amount of additional data is required to train multiple classifiers, significantly increasing training costs. During the inference phase, classifiers guide the diffusion model to generate specified types of data through gradients. Importantly, the performance of the classifiers affects the quality of the generated data, especially when the classifiers are not well-trained. However, Classifier-Free guidance does not have this limitation. Due to the integration of label information directly into the diffusion process through Classifier-Free Guidance, the model is able to effectively capture the latent distribution of specific types of data and dynamically adapt to multi-attribute scenarios.

**Figure 3:**
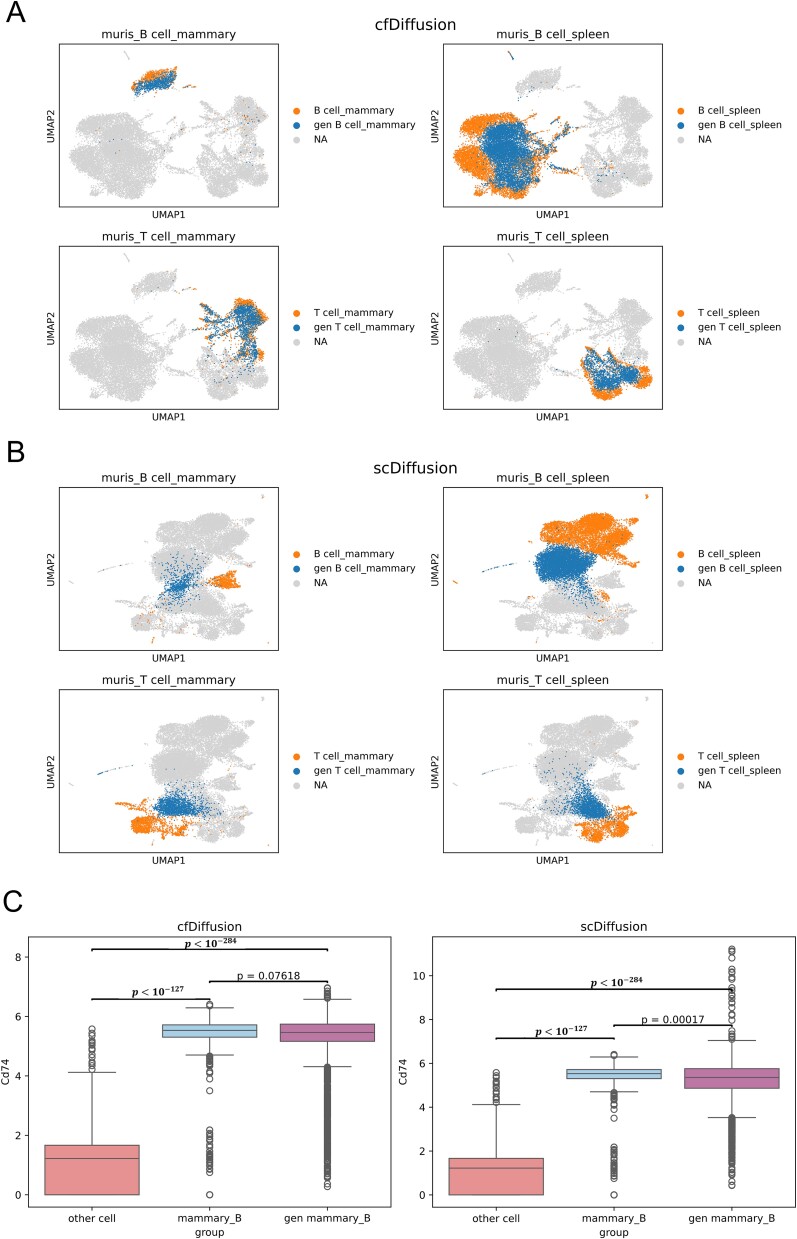
The quality assessment of simulated data by cfDiffusion and scDiffusion under multiple conditions is compared. (a) and (b) present UMAP visualizations of the simulated data for mammary T cells, mammary B cells, splenic T cells, and splenic B cells using the two methods. (c) and (d) depict box plots illustrating the distribution of Cd74 marker gene expression in simulated mammary B cell data. In these plots, ‘gen mammary_B’ represents the simulated gene expression data for mammary B cells generated by the models, ‘mammary_B’ denotes the real gene expression data for mammary B cells, and ‘other cell’ refers to the real gene expression data for mammary T cells, splenic T cells, and splenic B cells. Outliers in the distribution are indicated by circles. Finally, the Wilcoxon rank-sum test is employed to calculate the p-values between each pair of data groups.

The muris_mam_spl_T_B dataset consists of single-cell data with two attributes: organ type (mammary gland or spleen) and cell type (T cell or B cell). The dataset is composed of mammary T cells, mammary B cells, spleen T cells, and spleen B cells. During the training of cfDiffusion, we used both organ type and cell type as paired labels to train the Diffusion model. In the inference stage, we set the skipping step size of cfDiffusion to 5 and generated specific single-cell gene expression data by guiding the Diffusion model with label information. We combined the organ type and cell type labels to generate data for four specific labels: mammary T cells, mammary B cells, spleen T cells, and spleen B cells. The UMAP visualization (Fig. 3a, b) shows that most of the generated data covers the real data, with some generated data scattered around the real data. This indicates that the model has learned the attribute features of organ type and cell type. We selected the marker gene Cd74 for mammary B cells and plotted the expression distribution of Cd74 in real cells (excluding mammary B cells), real mammary B cells, and generated mammary B cells referenced scDiffusion. The Wilcoxon rank-sum test yielded a p-value of 0.07618 for the comparison between the generated mammary B cell data and the real mammary B cell data, indicating no significant difference between the two distributions. In contrast, the p-value for the comparison between the scDiffusion-generated mammary B cell data and the real mammary B cell data was 0.00017, indicating a significant difference between the two distributions. Unlike scDiffusion, which requires training two classifiers for organ type and cell type, respectively, cfDiffusion does not require training any classifiers, resulting in significant savings in training costs. As shown in Fig. 3b and Supplementary Table 3, the distribution of single-cell data generated by scDiffusion deviates more significantly from the real data distribution, indicating that scDiffusion performs less well than cfDiffusion in multiple conditions. In this experiment, the performance of scDiffusion in generating multi-attribute single-cell data was significantly lower than that observed in Experiment 3.1, where single-attribute single-cell data was generated. This indicates that the two classifiers were unable to effectively guide the diffusion process during the inference stage.

**Figure 4:**
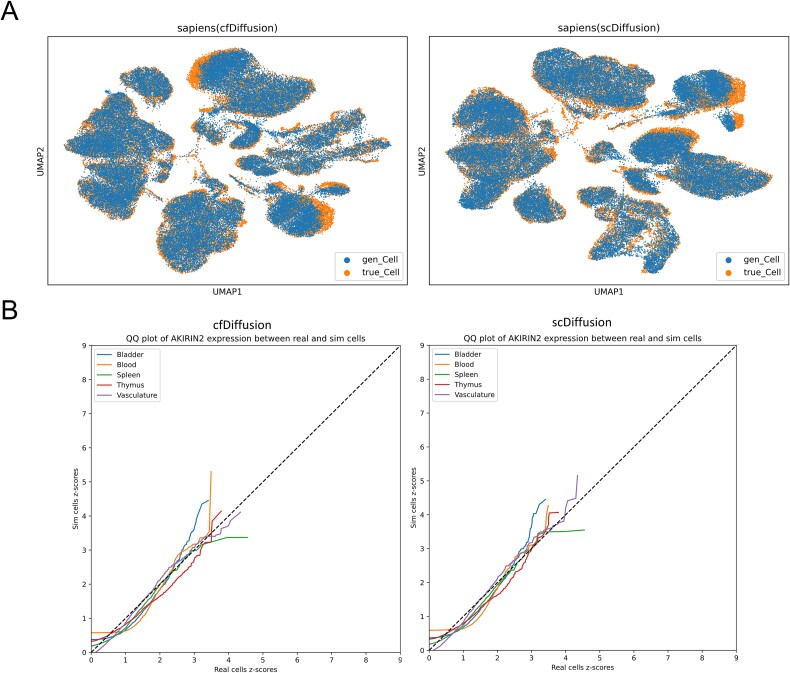
Quality assessment of cfDiffusion and scDiffusion simulated data under combined conditions. (a) UMAP visualization of simulated sapiens data generated by the two methods. (b) QQ-plot comparing the expression of the AKIRIN2 gene in five organs (bladder, blood, spleen, thymus, and Vasculate) between simulated and real data, with five colors corresponding to the expression in each organ, and the dashed line *y = x* serving as a reference line, where the trend of the lines should approximately follow the reference line if the two datasets have identical distributions.

In the Sapiens dataset, single-cell data possesses two attributes, differing from the muris_mam_spl_T_B dataset in that the organ type and cell type do not always correspond one-to-one. Therefore, we combined multiple conditions, such as organ type ‘Bladder’ and cell type ‘fibroblast’, into a single condition, denoted as ‘fibroblast_Bladder’. We trained both cfDiffusion and scDiffusion separately using this approach and visualized the simulated data. As shown in Fig. 4a, the distributions of the simulated data and real data generated by both methods are roughly similar. The QQ-plot (Fig. 4b) illustrates that the trends of the five colored lines, representing the expression distribution of the AKIRIN2 gene in five organs (Bladder, Blood, Spleen, Thymus, and Vasculate), are generally close to the reference line (dashed line), indicating that both methods simulate data with expression distributions similar to the real data. The simulation data generated by cfDiffusion under 16 labels are shown in Supplementary Fig. 4. The quality of the simulated data generated by cfDiffusion and scDiffusion is evaluated in Supplementary Table 4 and Table 5, respectively. Under various evaluation metrics, cfDiffusion outperforms scDiffusion almost entirely. The experiment demonstrates that cfDiffusion exhibits good stability in performance across datasets of the same cell type from different tissues.

### Comparison of scRNA-seq data generation quality across multiple platforms

We compared cfDiffusion (using a jump step of 5 during inference) with four existing generative models, scDiffusion, cscGAN, LSH-GAN, and sciGAN, using SCC, PCC, Wasserstein, MMD, and ILISI metrics to evaluate the quality of the single-cell gene expression data generated by these models. We also employed UMAP visualization to depict the single-cell gene expression data. For this experiment, we selected the Alles, Baron_Human, Marshall, and Mizrak datasets. Alles and Mizrak were generated using Drop-seq sequencing technology, Baron_Human was generated using InDrop sequencing technology, and Marshall was generated using 10X sequencing technology. These are all mainstream technologies or platforms in the field of RNA sequencing, and they have their own applications in different scenarios. We selected RNA datasets under different sequencing technologies to demonstrate that cfDiffusion can simulate single-cell data under various sequencing technologies and has certain generalization capabilities. The four datasets encompass different cell types, allowing us to assess each model's robustness in generating single-cell gene expression data across various scenarios.

Supplementary Table 6, Supplementary Table 7, Supplementary Table 8, and Supplementary Table 9 present the performance of the five models across the five evaluation metrics. Our method, cfDiffusion, outperformed the others on the Baron_Human, Marshall, and Mizrak datasets, and achieved the highest ILISI score on the Alles dataset. The scores for the remaining metrics were comparable to those of scDiffusion, with only minor differences. Notably, the MMD and ILISI metrics indicated that the single-cell gene expression data generated by cfDiffusion had a smaller distribution difference and higher integration with the real single-cell gene expression data.

**Figure 5:**
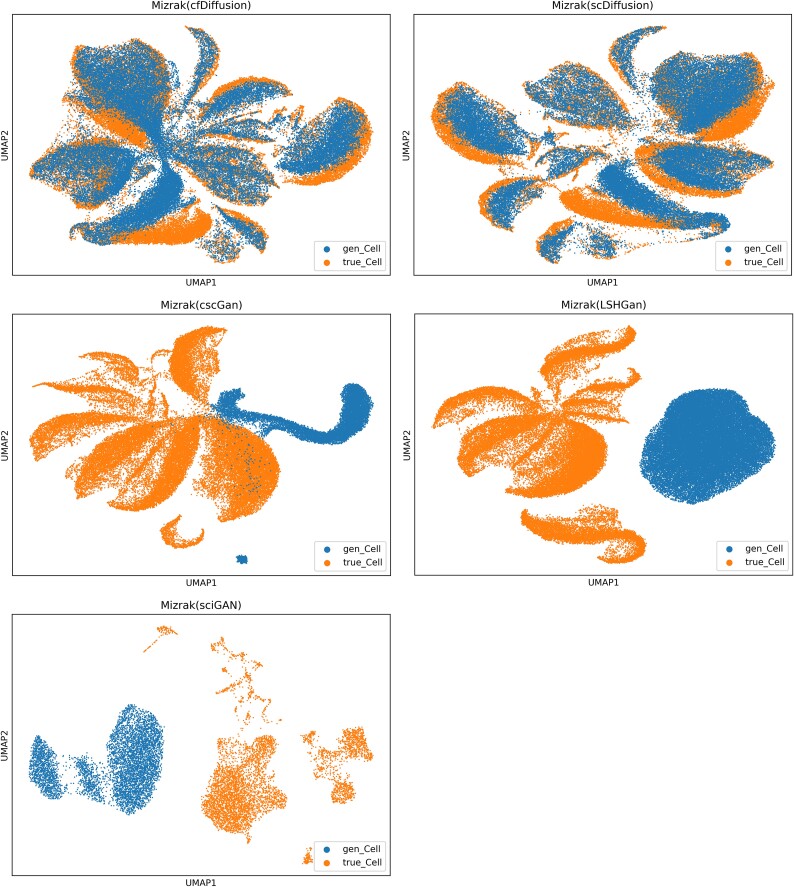
UMAP visualization of the distribution of simulated Mizrak dataset generated by five methods. The ‘gen_cell’ designation represents the distribution of the simulated data, while ‘true_cell’ indicates the distribution of the real data; a closer alignment between gen_cell and true_cell is preferable.

Furthermore, we used UMAP visualization to depict the high-variance genes, as shown in Supplementary Fig. 6, Supplementary Fig. 7, Supplementary Fig. 8 and Fig. 5 (combined with performance metrics Supplementary Table 6, Supplementary Table 7, Supplementary Table 8, and Supplementary Table 9, respectively). The distributions of the single-cell gene expression data generated by cfDiffusion and scDiffusion were closer to those of the real single-cell gene expression data, while the distributions of the data generated by the other methods deviated more significantly from the real single-cell gene expression data distributions.

### Simulating pseudo-time data

Cell trajectory analysis typically employs dimensionality reduction and clustering techniques to identify cellular heterogeneity and reconstruct potential developmental paths. By elucidating how cells transition between developmental stages, cell trajectory analysis provides insights into normal biological processes and pathological conditions. We applied it to Wadding-OT dataset.

The Wadding-OT dataset captures the differentiation states of induced pluripotent stem cells (iPSCs) over 18 days, with a sampling interval of 0.5 days, totaling 254,197 cells, and retaining 19,423 genes. Due to the massive size of the dataset, we selected 15 time points, including Day 0, Day 0.5, Day 1, Day 1.5, Day 2, Day 2.5, Day 3, Day 4.5, Day 5, Day 5.5, Day 6, Day 6.5, Day 7, Day 7.5, and Day 8, resulting in 82,920 cells. We used these time points as labels for the gene expression data and trained cfDiffusion with paired gene expression data and labels. During the inference phase, we set the jump step of cfDiffusion to 5. For each time point, we generated 5600 cell states and evaluated the generated data using various metrics, as shown in Supplementary Tables 10 and 11.

**Figure 6:**
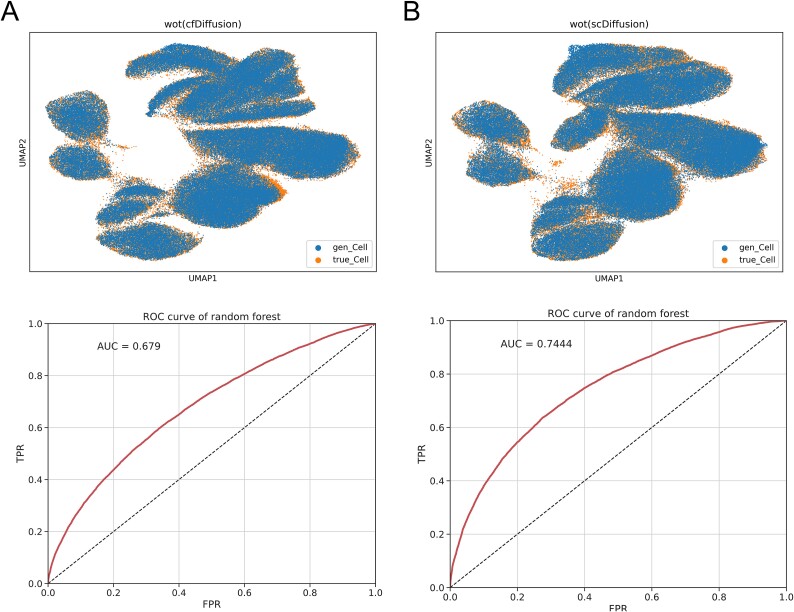
UMAP visualization of WOT data distributions simulated by two methods and random forest ROC curves. (a) Represents the results using the cfDiffusion method, and (b) represents the results using the scDiffusion method.

In the UMAP dimensionality reduction and visualization (as shown in Fig. 6), we observed that the generated data and real data distributions were largely similar, with SCC and PCC values exceeding 0.99, indicating that the gene expression patterns of the generated single-cell data were extremely similar to those of the real single-cell data. The ILISI value of 0.8198 represents that the generated single-cell data is essentially homogeneously mixed with the real single-cell data. Additionally, the random forest model showed no overfitting during training, testing, and AUC evaluation (as shown in Fig. 6 and Supplementary Fig. 3), suggesting that the generated data were highly similar to the real data, making it difficult to distinguish between them. We trained K-nearest neighbors classifiers using the real data and model-generated data at different time points, and the average prediction accuracy was approximately 0.5, further highlighting the high quality of the single-cell data generated by cfDiffusion at each time point.

Comparing cfDiffusion and scDiffusion across various evaluation metrics, we observed that cfDiffusion outperformed scDiffusion in terms of MMD, ILISI, and random forest evaluation results, while the other metrics showed only minor differences or equivalency. This suggests that cfDiffusion performs better than scDiffusion to a certain extent.

We applied the Leiden algorithm to cluster the data generated by cfDiffusion, scDiffusion, and the original data, with the clustering results shown in Supplementary Fig. 11. Based on three clustering metrics (ACC, ARI, NMI), the performance of cfDiffusion simulated data was the best, indicating that cfDiffusion effectively learns the feature distributions of different cell types, making the generated data more amenable to clustering. Additionally, after dimensionality reduction using UMAP, cfDiffusion retained the biological topological structure of the original data (as shown in Supplementary Fig. 9).

To further explore the application of cfDiffusion-generated data in downstream analysis tasks at the single-cell level, we focused on pseudotime analysis. Pseudotime is a crucial tool for reconstructing cellular dynamics, encompassing differentiation pathways, developmental timelines, and disease trajectories. In this experiment, we used the Slingshot [34] algorithm to analyze the reprogramming process of fibroblasts to iPSCs. Slingshot can construct cellular differentiation lineages and infer pseudotime from single-cell RNA-seq data, utilizing cell clustering and spatial dimensionality reduction information to learn the relationships between cell clusters in an unsupervised or semi-supervised manner, revealing the global structure between cell clusters and converting this structure into a smooth lineage represented by a one-dimensional variable, referred to as ‘pseudotime.’

We selected single cells from the WOT dataset that underwent differentiation over an 8-day period and generated these single-cell data using both cfDiffusion and scDiffusion methods. The states of cell differentiation from Day 0 to Day 8 are represented as D0 to D8. We employed the Slingshot algorithm to calculate the differentiation trends of the simulated data. In Supplementary Fig. 9, the shading indicates the duration of differentiation, with the lightest color representing D0 and the darkest color representing D8. From Supplementary Fig. 9, we can observe that the pseudo-time inference for the cfDiffusion simulated single-cell data is approximately: D0 - > D0.5 - > (D2, D2.5, D3) - > (D4.5, D5, D5.5) - > D8 - > (D6, D6.5, D7.5), and D0 - > D0.5 - > (D1, D1.5). The true pseudo-time inference is: D0 - > D0.5 - > D1 - > D1.5 - > D2 - > D2.5 - > D3 - > D4.5 - > D5 - > D6 - > D6.5 - > D7 - > D7.5 - > D8. Aside from some discrepancies at the ‘D8’ time point compared to the true differentiation trajectory, the movement trajectories of the other cells are similar to the actual trajectories. In contrast, the pseudo-time inference from the scDiffusion simulated data shows minimal alignment with the true differentiation trajectory (see Supplementary Fig. 10). This experiment demonstrates that cfDiffusion simulated data possesses a degree of reliability in complex biological explorations, such as cell trajectory and differentiation analysis.

Furthermore, to simulate varying degrees of noise, we randomly set 20%, 40%, and 60% of the gene expression values of each cell in the Wadding-OT data to zero (a process referred to as ‘dropout’) to test the robustness of the cfDiffusion model. The experimental results are presented in Supplementary Figs. 12 and 13, indicating that cfDiffusion can tolerate dropout rates of up to 20%. However, as the sparsity of the data increases, the loss of cell type features becomes more pronounced, resulting in the model's inability to effectively learn the features.

## Discussion

scDiffusion is a Classifier Guidance-based model designed for generating single-cell data of specified types. At each inference timestep, the model requires the computation of classifier gradients to guide the diffusion process. However, training the classifier requires a large amount of noisy data. When single-cell data exhibit multiple attributes, an equivalent number of classifiers must be trained, which significantly increases the model's training cost. Moreover, the increased gradient computation during inference impacts the model's efficiency. Additionally, the quality of the classifier directly affects the generated results according to specific categories, as demonstrated in the experimental results of Section 3.2. Therefore, Classifier Guidance methods are less suitable for applications involving data with multiple attributes. However, they are suitable for scenarios where data attributes change or the number of categories is uncertain. In such cases, only the classifier needs to be retrained, without the need to retrain the entire diffusion model. In contrast to Classifier Guidance, Classifier-Free Guidance does not require a classifier for guidance, yet is still capable of generating data of specified types. It is particularly suitable for data with multiple attributes and in situations where hardware resources are limited.

To address the limitations of scDiffusion, we propose an improved scRNA-seq data generation model based on the denoising diffusion probabilistic model structure, named cfDiffusion. This model employs a Classifier-Free approach to generate multi-condition single-cell data without the need for classifier-guided gradients, significantly reducing training costs and simplifying the training procedure compared to scDiffusion. During the inference phase, we introduce a mechanism for caching high-level features, which enables the model to rapidly generate single-cell gene expression data while ensuring data quality by setting different jump step sizes. We benchmark cfDiffusion against other generative models: cscGAN, LSH-GAN, sciGAN, and scDiffusion. The primary goal of these four methods is to generate high-quality data, with cscGAN and sciGAN capable of generating gene expression data under single conditions only, while LSH-GAN can only generate unconditional gene expression data. Although scDiffusion can generate gene expression data with multiple attributes, it requires the additional training of multiple classifiers, and the performance of these classifiers can impact the quality of the generated data, as validated on the muris_mam_spl_T_B dataset. In comparison to these four methods, cfDiffusion can generate gene expression data with multiple attributes without requiring gradient guidance from classifiers. Furthermore, cfDiffusion demonstrates relatively stable performance, achieving the highest overall data quality across various benchmark tests. Experimental results also demonstrate that cfDiffusion can simulate single-cell data from various sequencing platforms, outperforming other methods in terms of data quality and exhibiting superior performance across multiple evaluation metrics. In experiments inducing stem cell differentiation into mouse embryonic fibroblasts, the simulated single-cell data at different time points closely resembled the distribution of real single-cell data. we have included the sparsity rates (i.e., the proportion of zeros in the gene expression matrix) for each dataset in Table 1. As detailed in Supplementary Tables 1–11, cfDiffusion consistently outperforms other methods across datasets of varying sizes and sparsity levels. Specifically, it demonstrates slightly better performance in datasets with lower sparsity rates (WOT, Baron) and larger dataset sizes (WOT). Importantly, even in datasets characterized by higher sparsity rates (Mizrak at 97.40% and PBMC68k at 96.94%) and smaller dataset sizes (Alles, Marshall, Baron), the performance of cfDiffusion remains relatively unaffected. Therefore, in future research, researchers can use cfDiffusion to simulate data reflecting different developmental stages or cell types [35], infer cell trajectories and lineage relationships between different cell types, explore complex biological systems [36], test hypotheses, and develop personalized therapeutic strategies. Theoretically, cfDiffusion can simulate any omics data, including but not limited to spatial transcriptomics, scATAC, proteomics, and DNA methylation data. We believe that with continuous iterations of Diffusion, it can be applied to other generative tasks, holding broader biological prospects and practical significance.

**Key Points**

- Integration of Classifier-Free Guidance Mechanism. The model innovatively incorporates a Classifier-Free Guidance approach in the diffusion framework for single-cell RNA sequencing data generation. This novel integration significantly reduces computational overhead during model training compared to traditional Classifier Guidance methods, while maintaining high fidelity in multi-attribute cellular data synthesis.
- Integration of High-Level Feature Caching Mechanism. This optimization strategy diminishes inference latency without compromising the model’s generative capabilities within small steps, thereby enhancing the practical applicability of the system in computational biology workflows.
- Compared to other methods, cfDiffusion demonstrates relatively stable performance in generating data from various sequencing platforms.

## Supplementary Material

Supplementary_Materials(Figures_and_Tables)_Final_bbaf071

Supplementary_Dataset_and_Model_bbaf071

## Data Availability

Tabular Muris, PBMC68k, Waddington-OT, *Homo Sapiens*, Human_PF_Lung, muris_mam_spl_T_B are available in figshare Dataset at (https://doi.org/10.6084/m9.figshare.25335436.v2). Alles, Baron, Marshall, Mizrak are accessible from Gene Expression Omnibus (Accession number: GSE89164, GSE84133, GSE109447, GSE158002). Source codes for the cfDiffusion python packages and the related scripts are available at (https://github.com/SuperheroBetter/cfDiffusion).
