# Supplementary material for: cfDiffusion: diffusion-based efficient generation of high quality scRNA-seq data with classifier-free guidance
Source: Brief Bioinform. 2025 Feb 23;26(1):bbaf071. doi: 10.1093/bib/bbaf071 (PMC11846686; doi:10.1093/bib/bbaf071)
Supplement: Supplementary_Materials(Figures_and_Tables)_Final_bbaf071 [file supplementary_materials(figures_and_tables)_final_bbaf071.pdf]

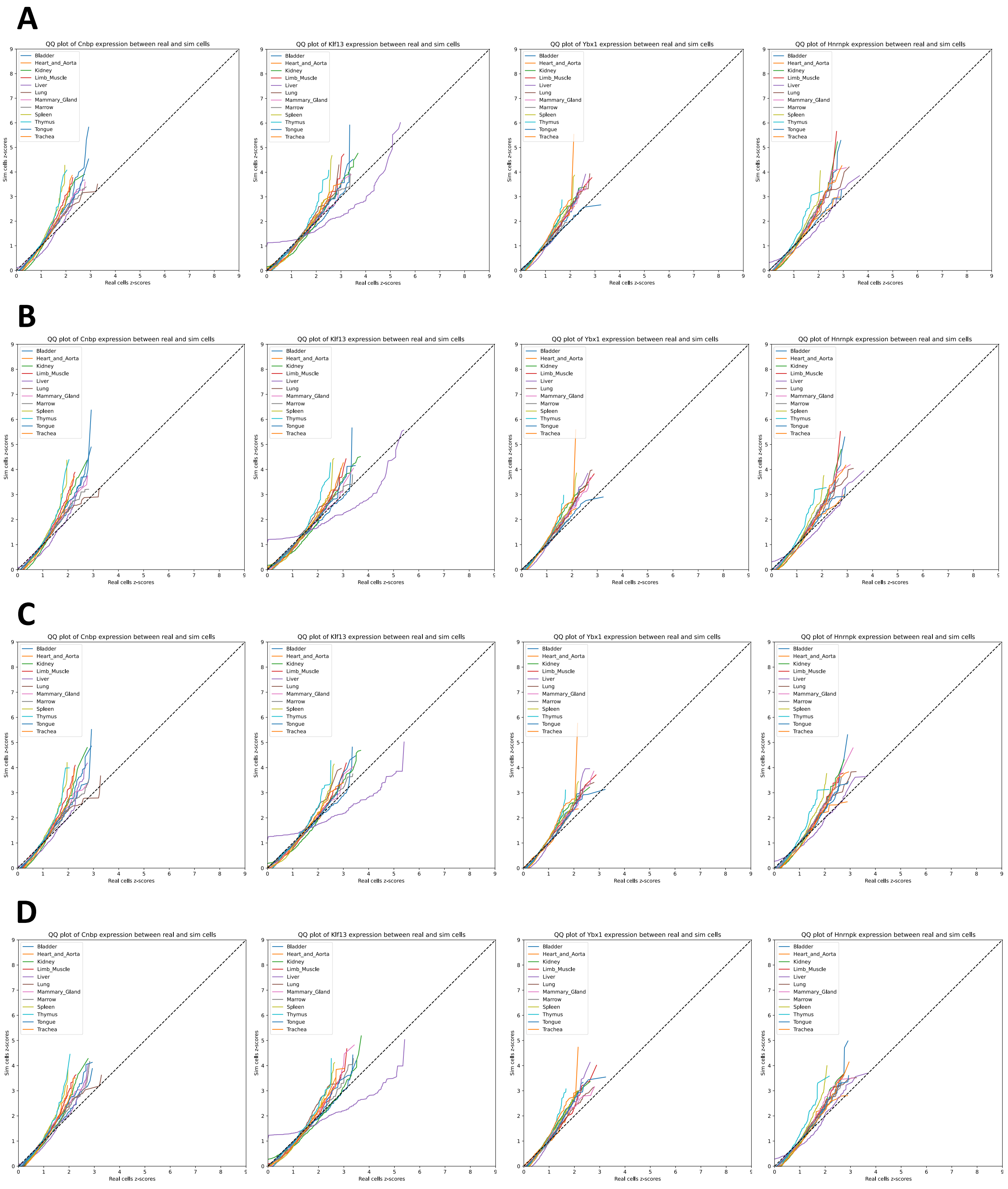

Supplementary Figure 1. Correlation analysis of marker genes between simulated and real Muris single-cell gene expression data at different skipping steps. (A) Skipping step = 5. (B) Skipping step = 10. (C) Skipping step = 20. (D) Skipping step = 50.

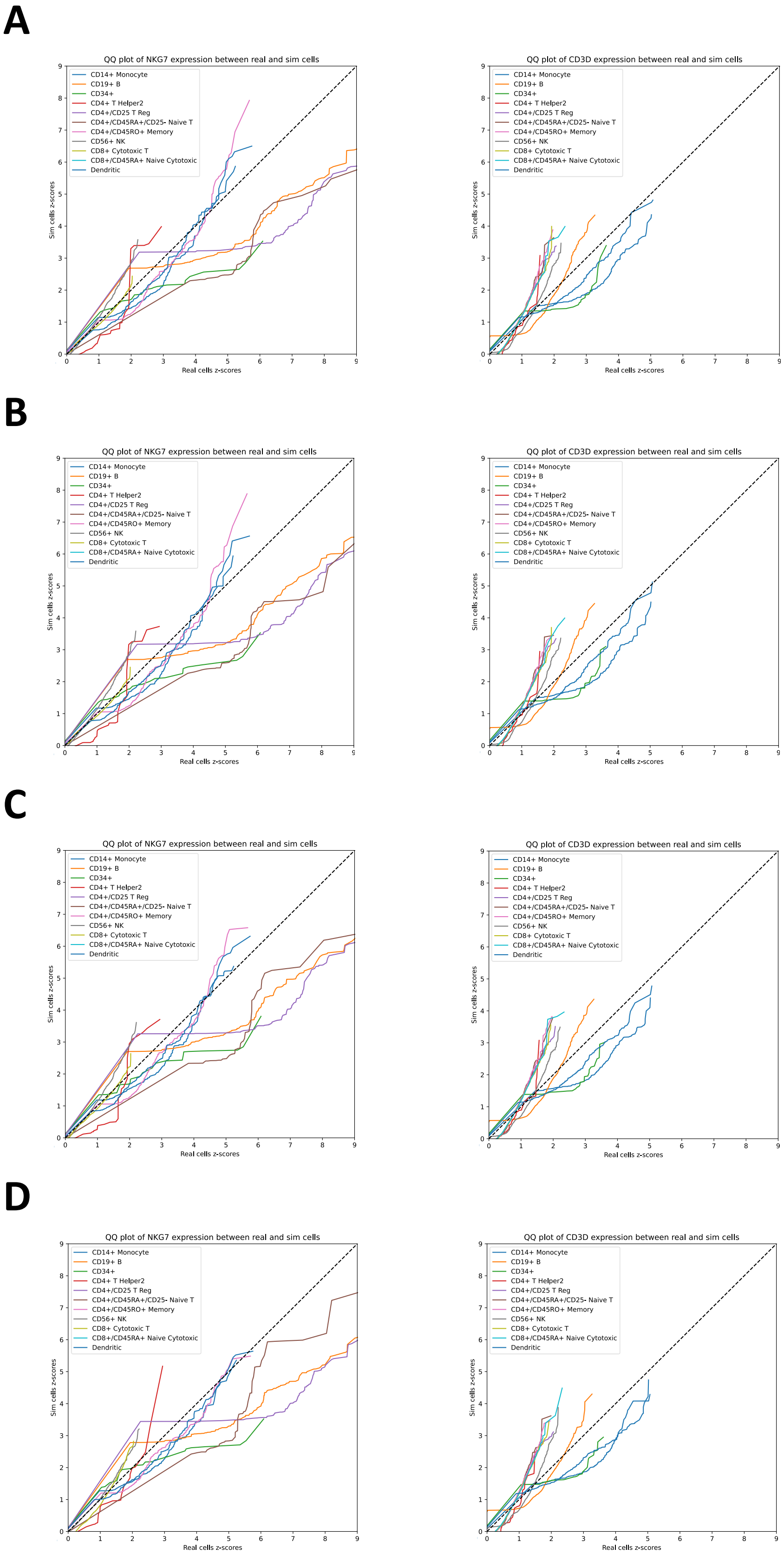

Supplementary Figure 2. Correlation analysis of marker genes between simulated and real Pbmc68k single-cell gene expression data at different skipping steps. (A) Skipping step = 5. (B) Skipping step = 10. (C) Skipping step = 20. (D) Skipping step = 50.

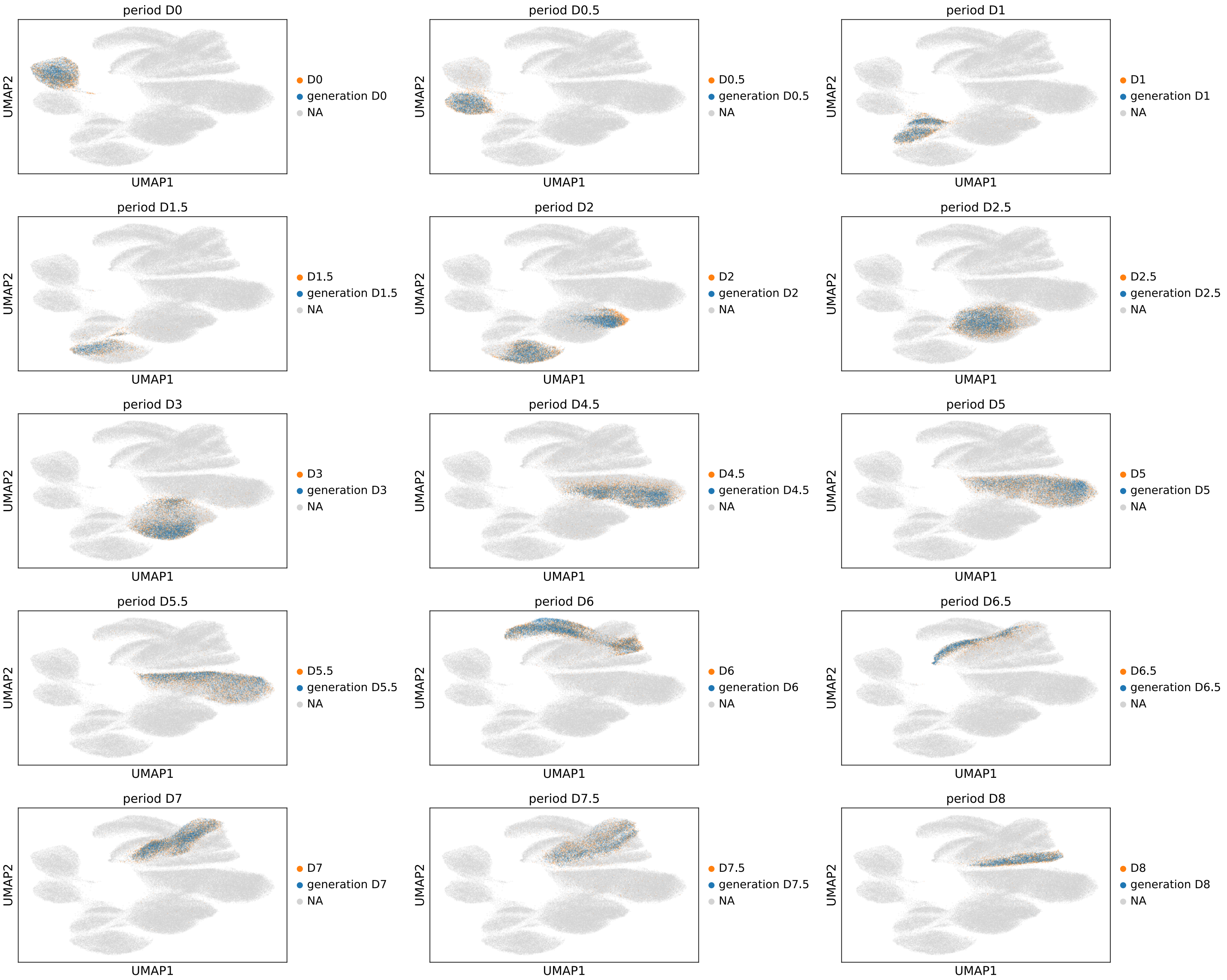

Supplementary Figure 3. Two-dimensional distribution of Waddington-OT simulated data generated by cfDiffusion on UMAP plots. D0-D8 represent days, depicting the differentiation and developmental states of mouse embryonic fibroblasts at different time points. Blue dots represent single-cell data simulated by cfDiffusion, while orange dots represent real single-cell data

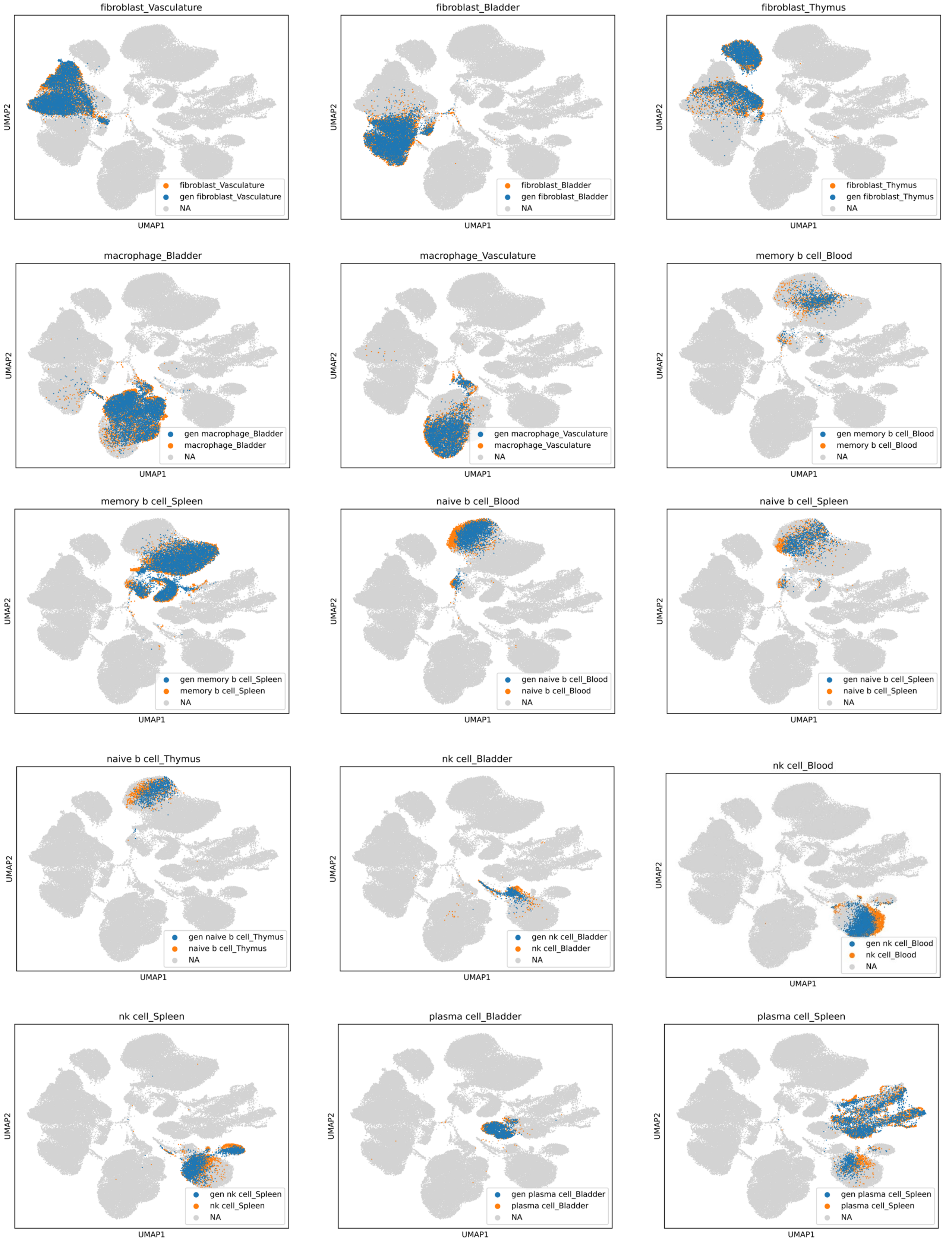

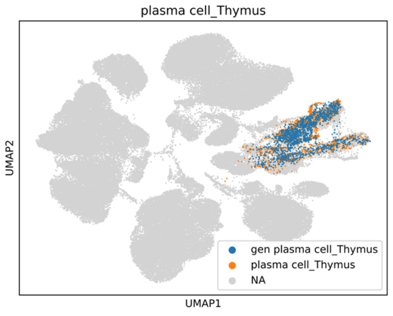

Supplementary Figure 4. Distribution of simulated data for 16 Homo Sapiens cell types generated by cfDiffusion on UMAP plots.

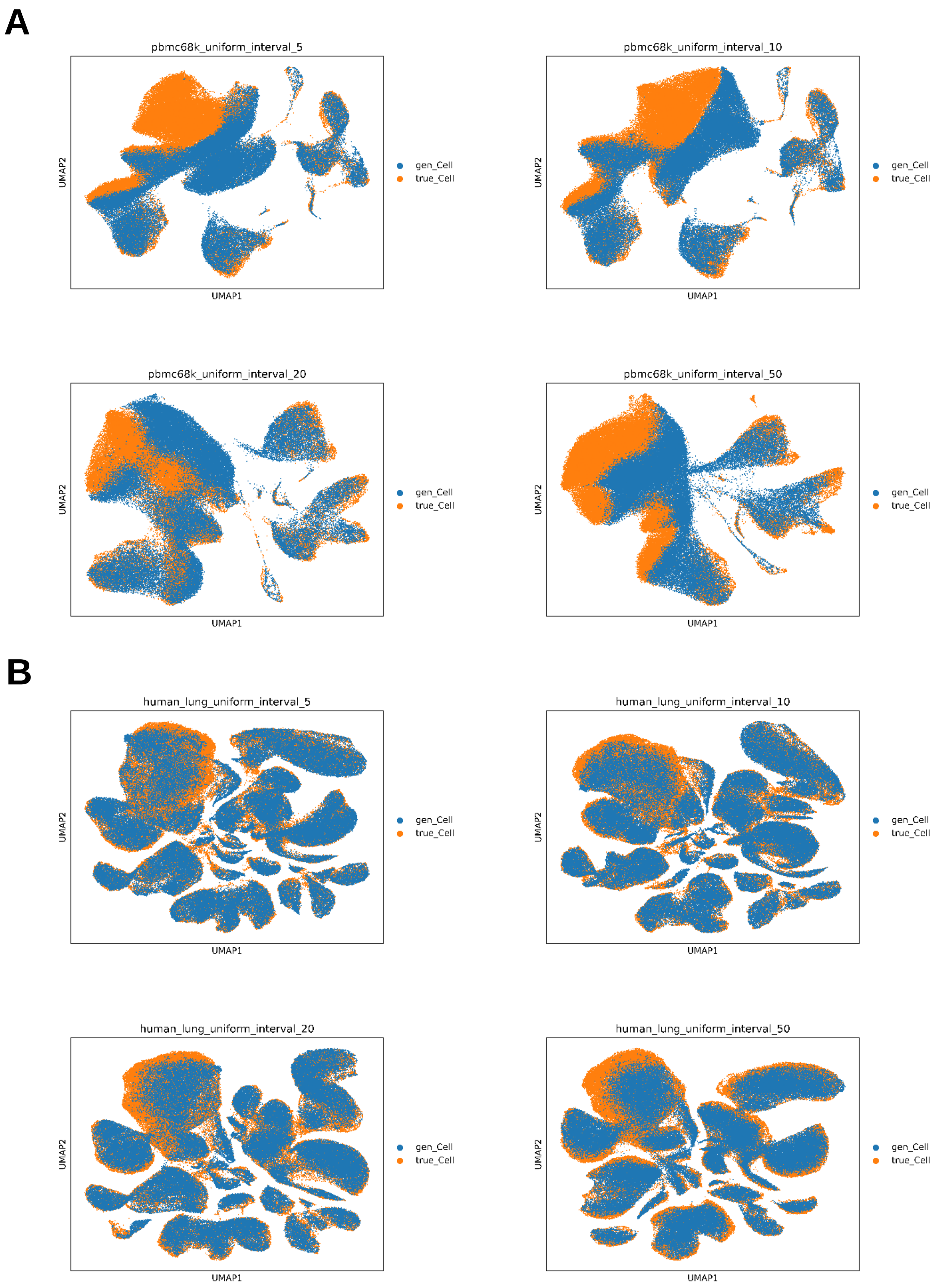

Supplementary Figure 5. UMAP visualization of real data and generated data in pbmc68k and human_lung datasets on a 2D space, showing changes in generated data quality when the model skipping step size is 5, 10, 20, and 50. (A) cfDiffusion-generated pbmc68k data with skipping steps of 5, 10, 20, and 50. (B) cfDiffusion-generated human_lung data with skipping steps of 5, 10, 20, and 50.

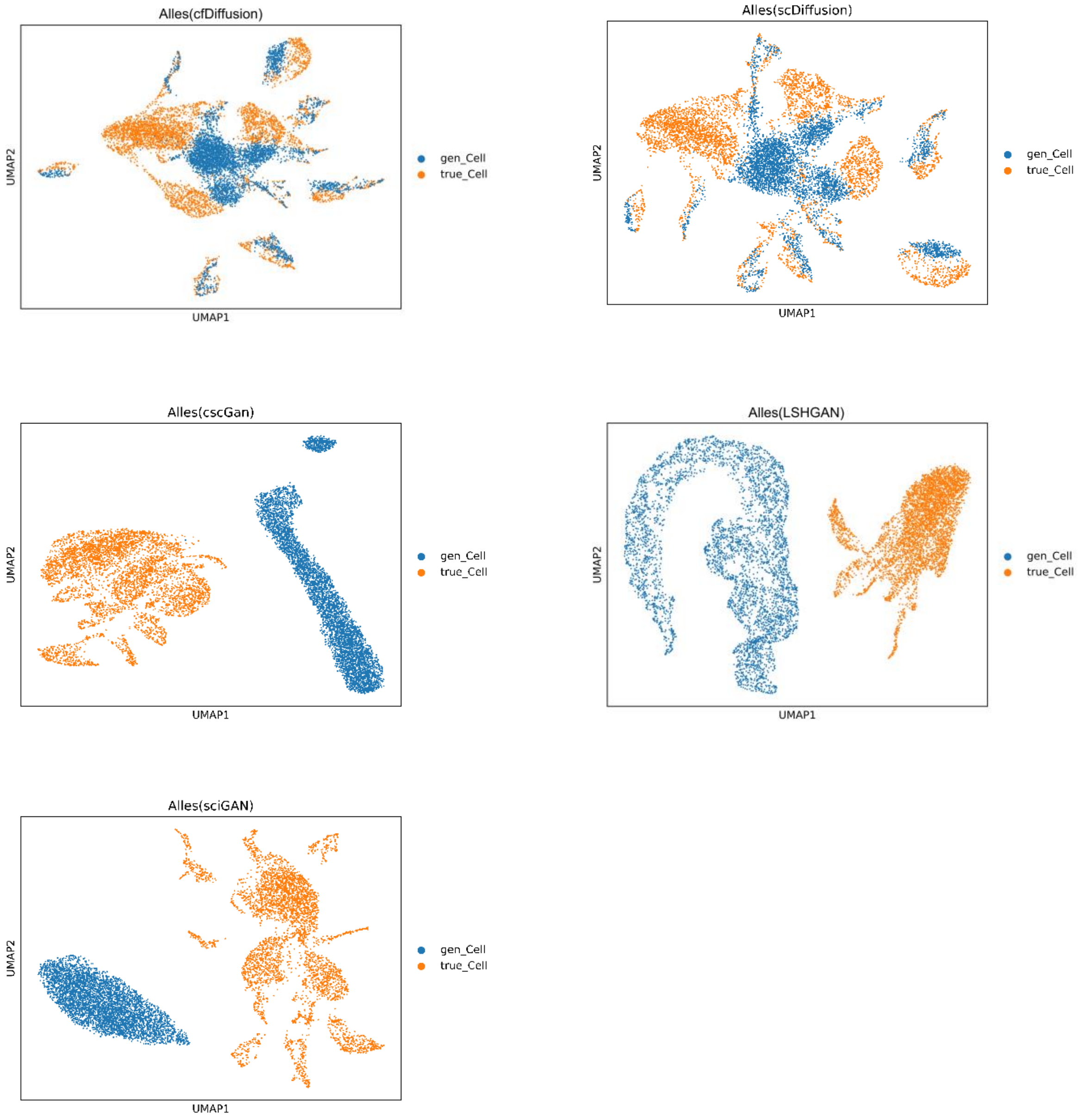

Supplementary Figure 6. UMAP visualization of the distribution of simulated Alles dataset generated by five methods.

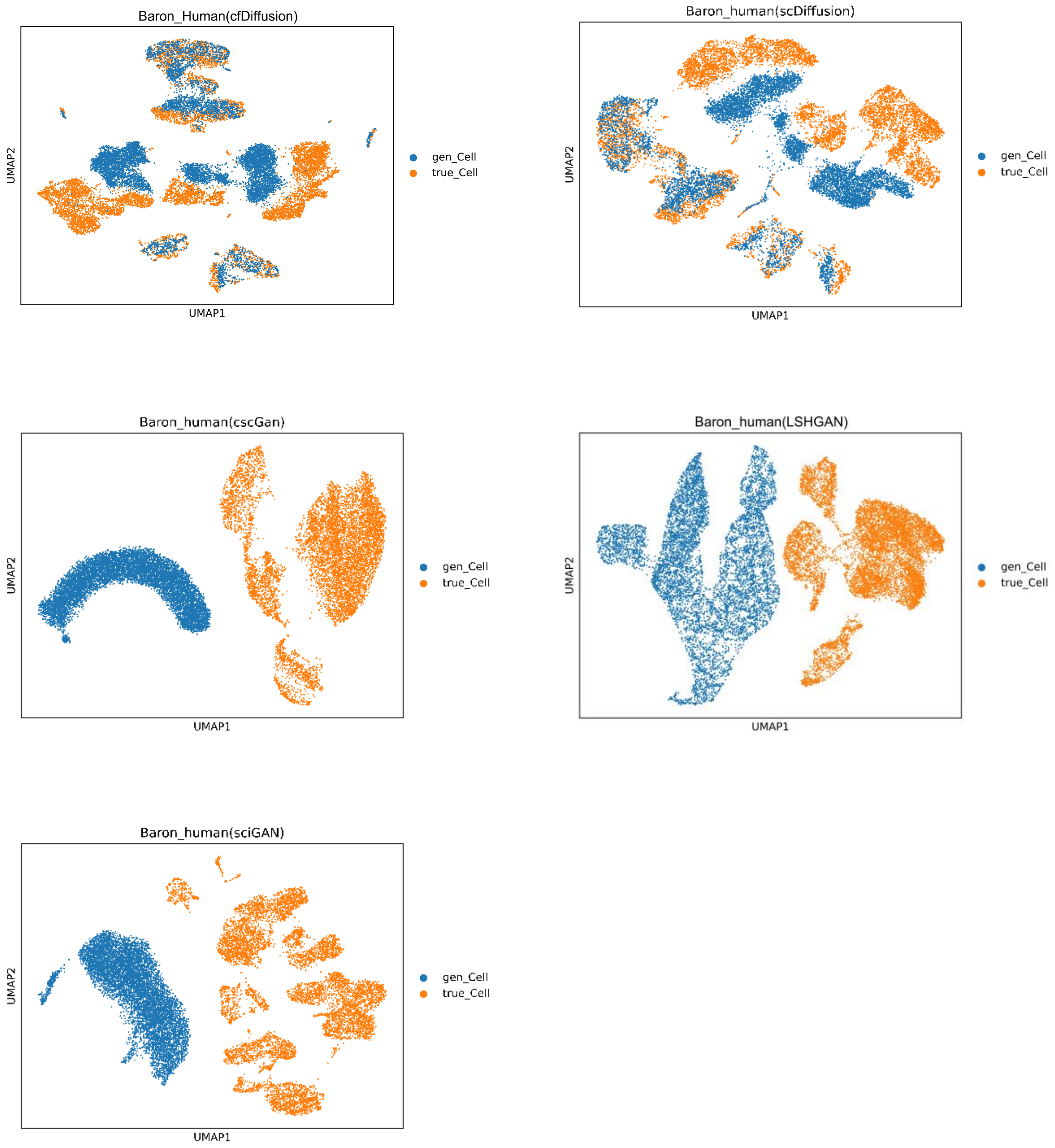

Supplementary Figure 7. UMAP visualization of the distribution of simulated Baron_Human dataset generated by five methods.

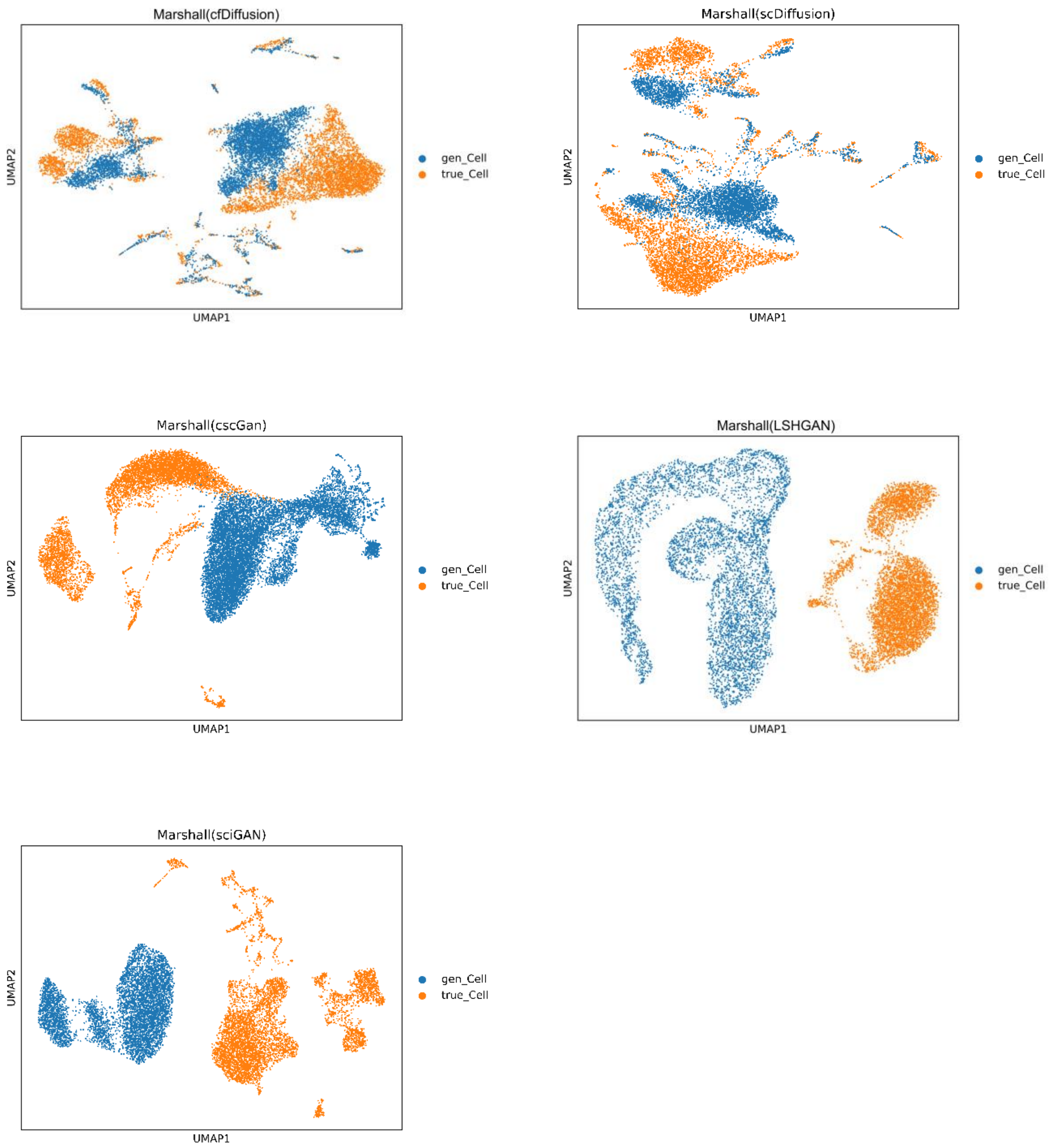

Supplementary Figure 8. UMAP visualization of the distribution of simulated Marshall dataset generated by five methods

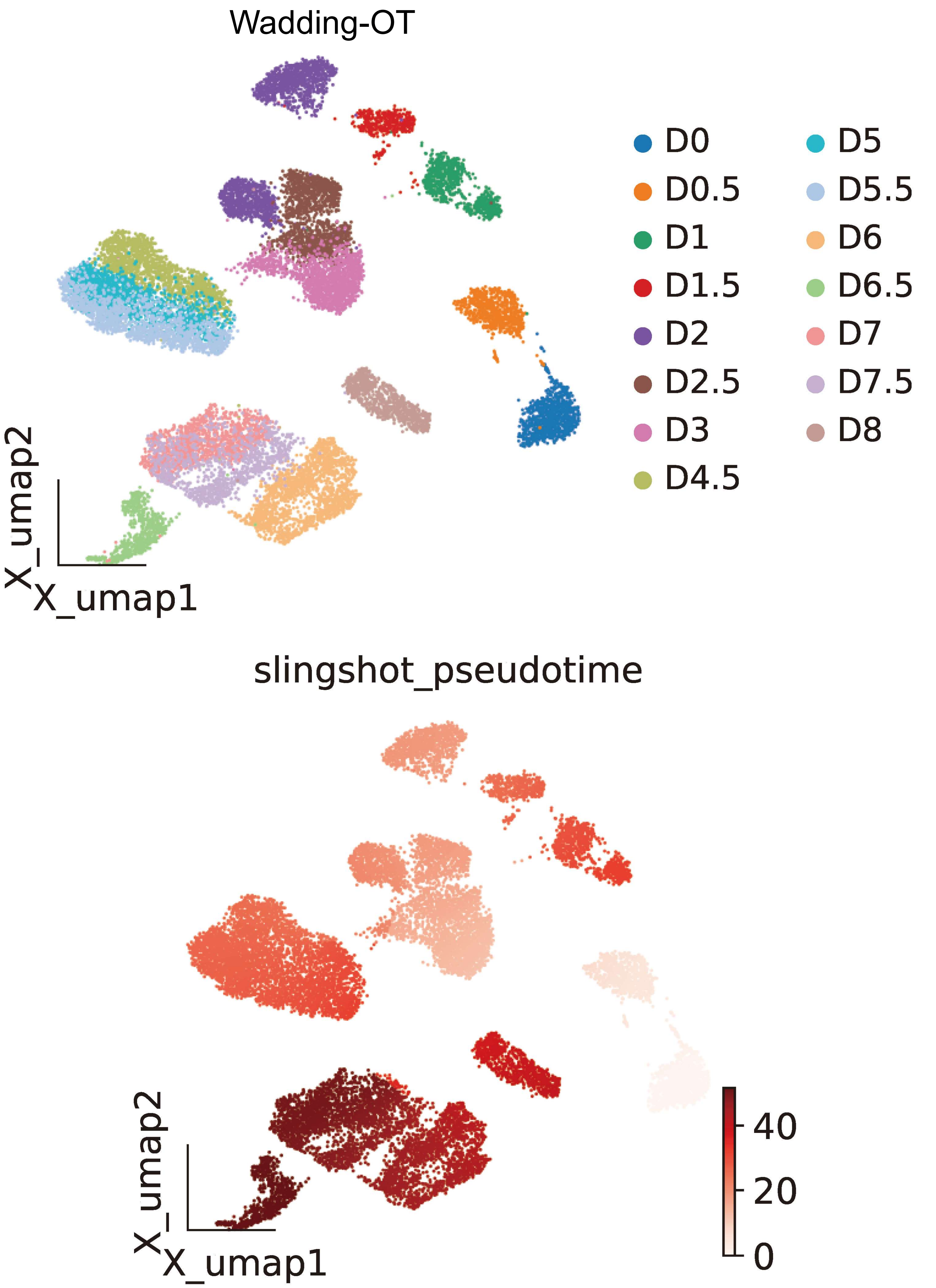

Supplementary Figure 9. Pseudo-time analysis of cfDiffusion simulated Washing-OT data

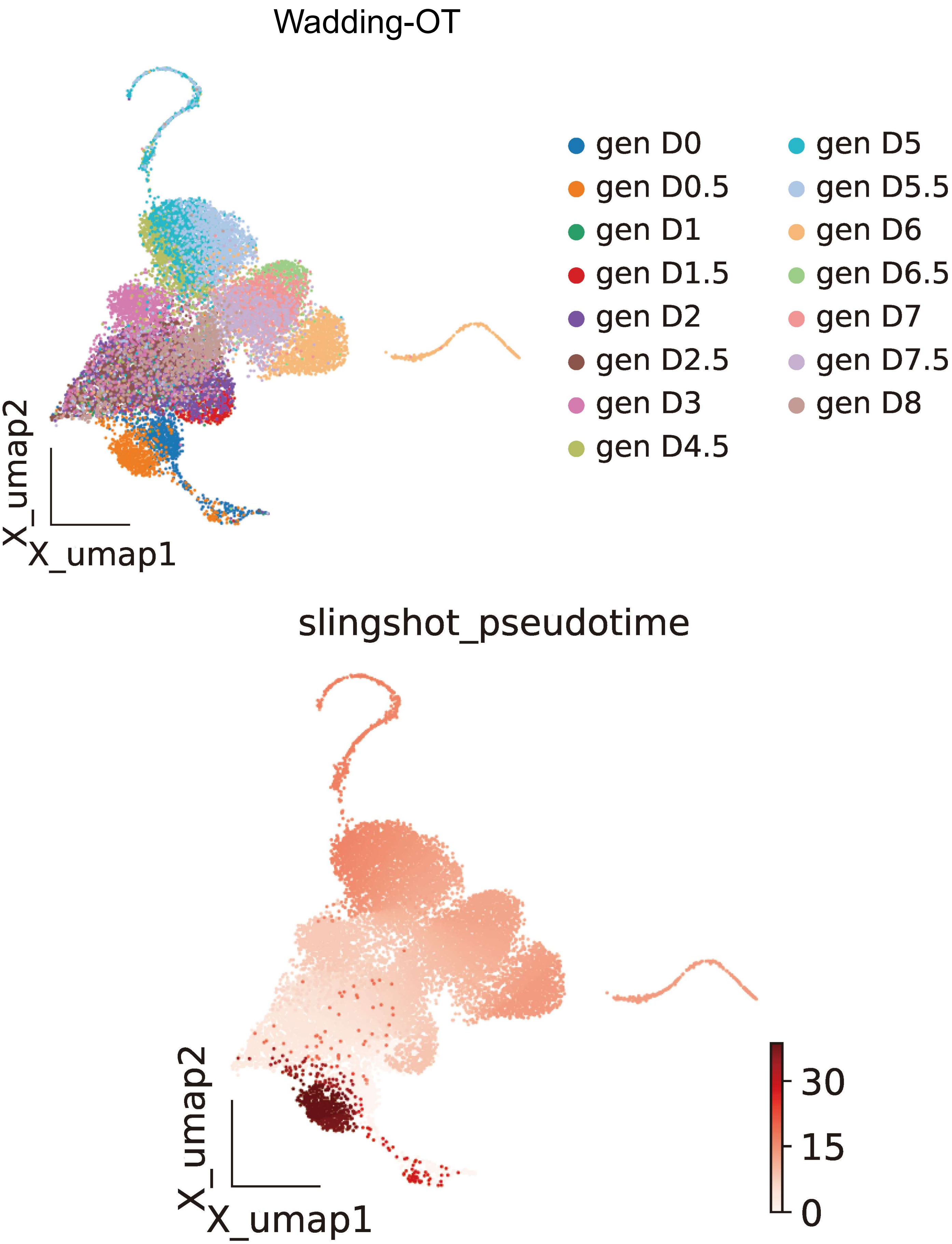

Supplementary Figure 10. Pseudo-time analysis of scDiffusion Washing-OT data

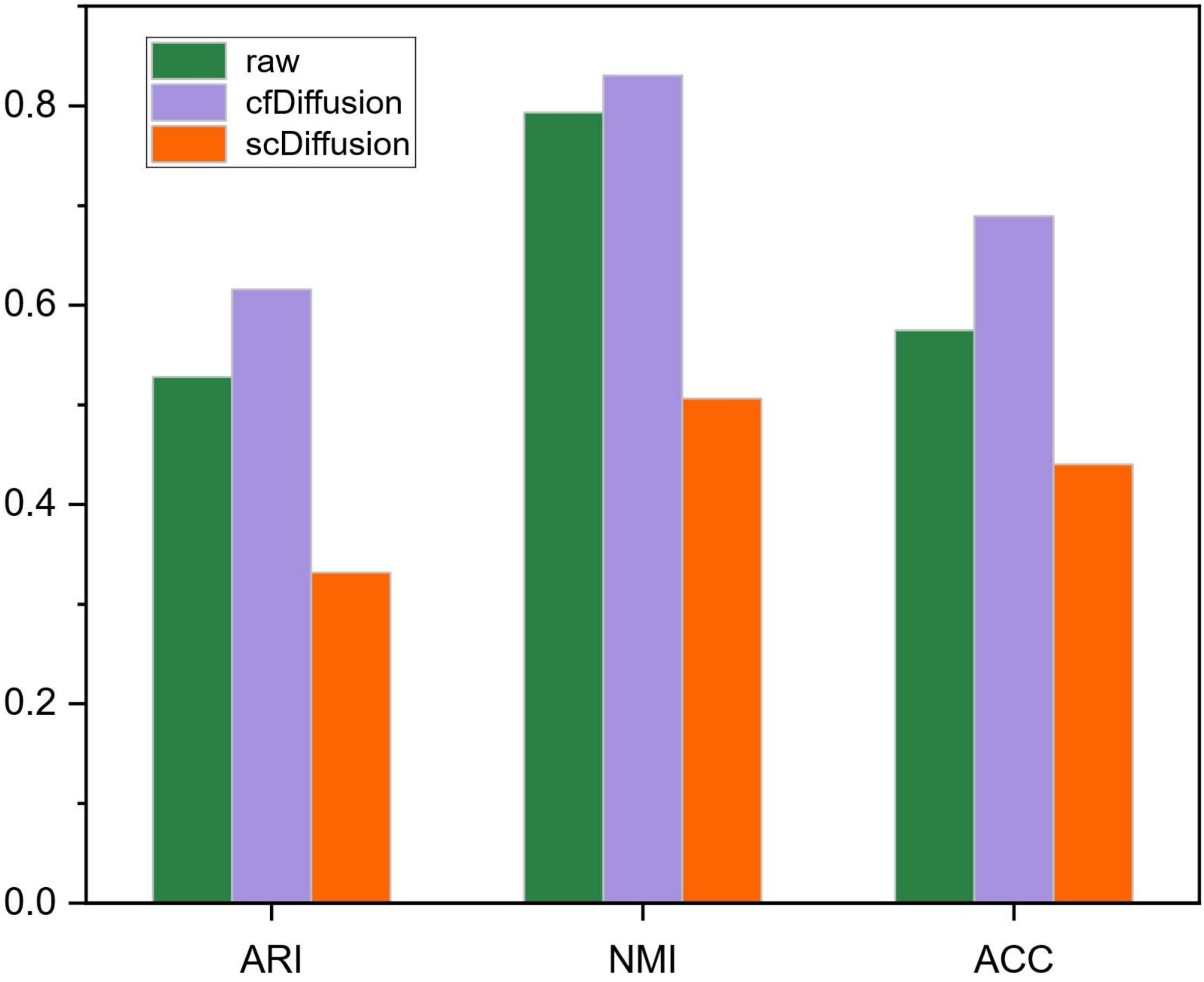

Supplementary Figure 11. Evaluation of raw data, cfDiffusion simulated data, and scDiffusion simulated data using ARI, NMI and ACC metrics.

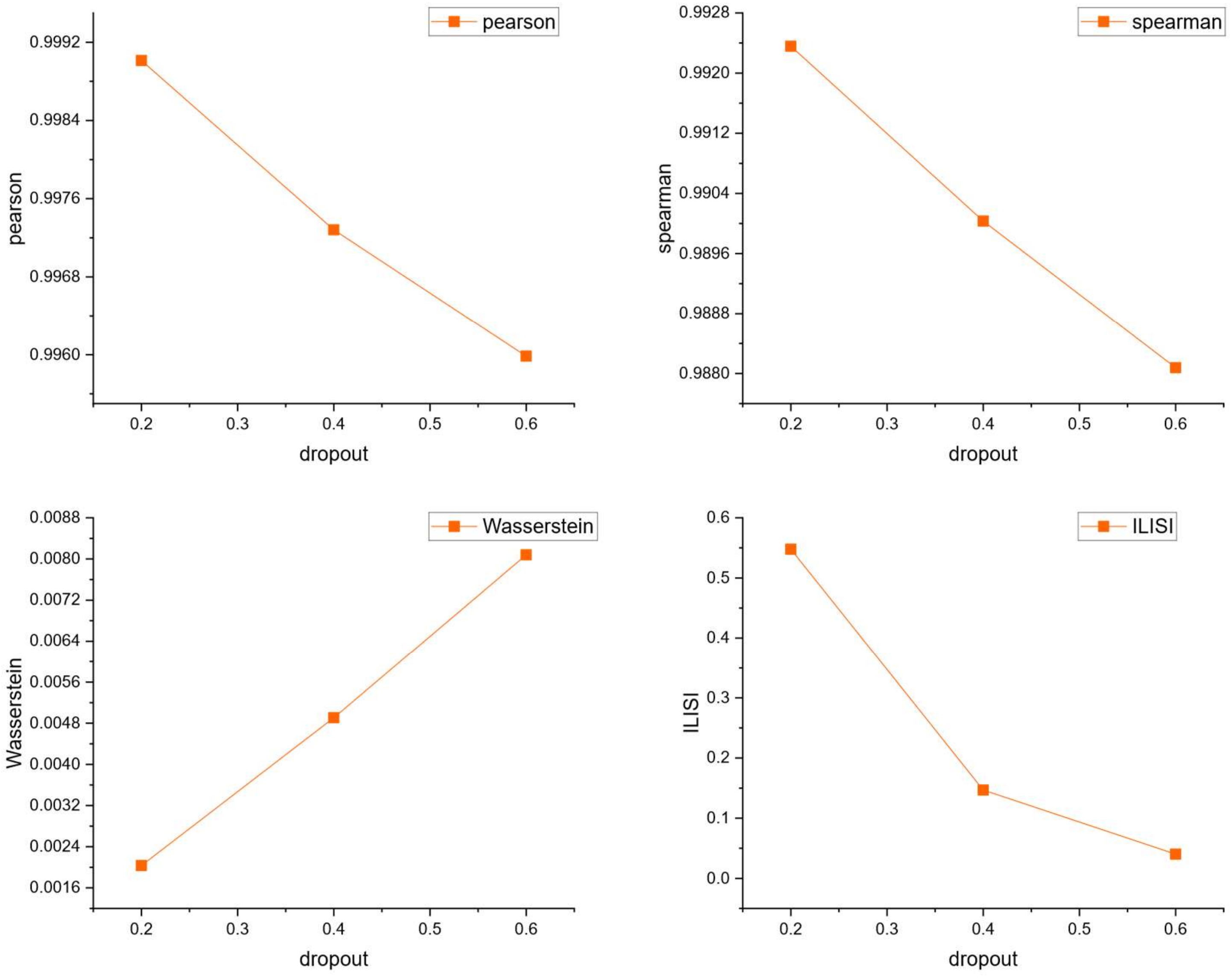

Supplementary Figure 12. Quality changes in cfDiffusion simulated data at 20%, 40%, and 60% dropout.

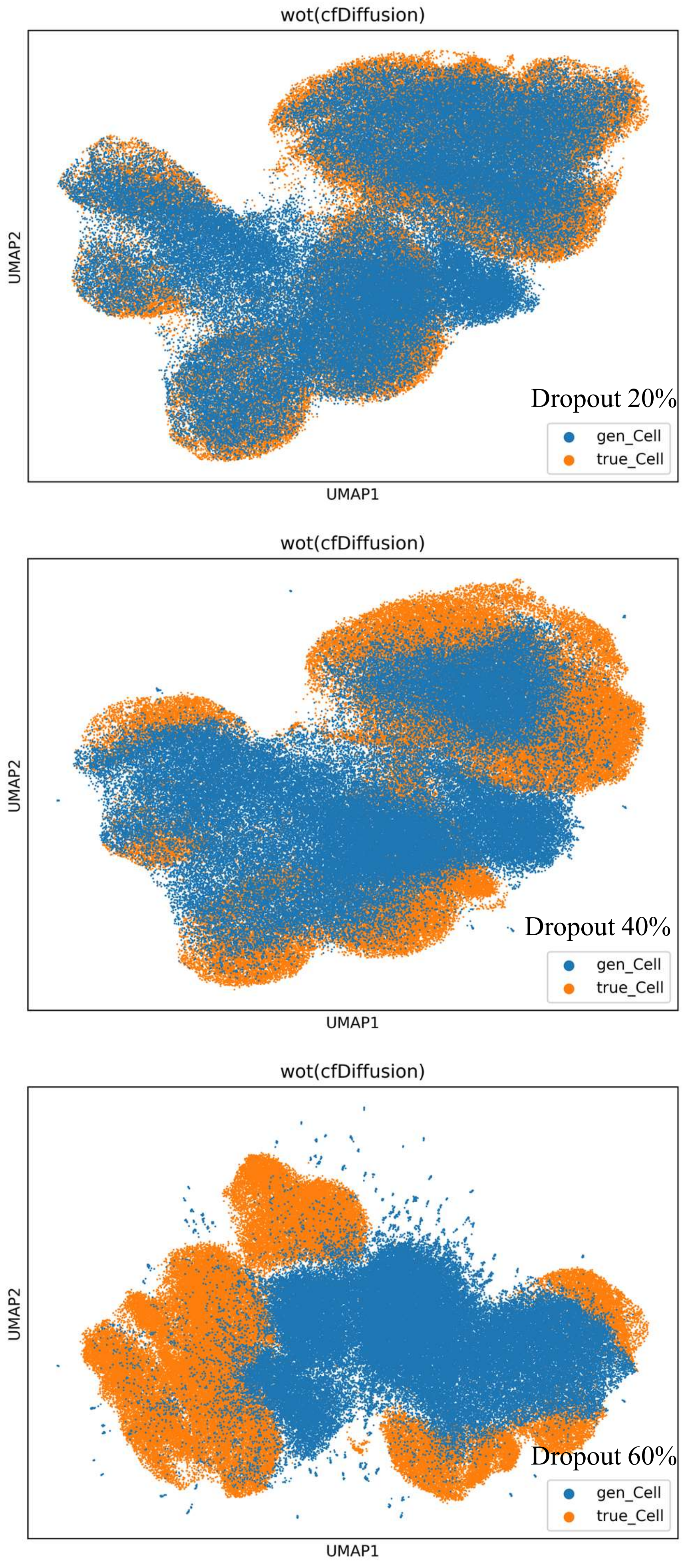

Supplementary Figure 13. UMAP of cfDiffusion simulated data at 20%, 40%, and 60% dropout.

scDifffusion											
Dataset	SCC	PCC	Wasserstein	MMD	ILISI	KNN_AUC	KNN_ACC	RandomForest_AUC	RandomForest_ACC_Train	RandomForest_ACC_Test	RandomForest_OOB_Score
sapiens	0.993978772	0.998778166	0.009288105	1.4049	0.769238863	0.5	0.498811066	0.8308	0.745044494	0.759067723	0.751173193
Human_PF_Lung	0.985558932	0.99796244	0.00554291	1.3459	0.28071277	0.502103771	0.49811475	0.8343	0.747442075	0.743298108	0.744428234
WOT	0.992612753	0.999703743	0.004577417	2.7052	0.792457603	0.507	0.499695247	0.7444	0.679112397	0.685021708	0.67895964
pbmc68k	0.957958655	0.998496804	0.006812875	0.5157	0.699111911	0.5	0.494412	0.798	0.724042633	0.713013931	0.717065546
muris	0.99206635	0.998843905	0.008905142	0.5753	0.580598094	0.5	0.497114397	0.7953	0.727313071	0.718480138	0.720428431

cfDiffusion step5											
Dataset	SCC	PCC	Wasserstein	MMD	ILISI	KNN_AUC	KNN_ACC	RandomForest_AUC	RandomForest_ACC_Train	RandomForest_ACC_Test	RandomForest_OOB_Score
sapiens	**0.994052985**	0.998764096	0.00927498	**1.3597**	**0.794556845**	0.509732701	0.498662918	**0.8143**	0.745146355	**0.734639107**	**0.736059013**
Human_PF_Lung	**0.988044131**	0.997912395	**0.005100234**	**0.57**	**0.55280687**	0.500855811	0.498258119	**0.7894**	**0.693964266**	**0.685968604**	**0.690344116**
WOT	0.98569443	0.99331667	0.01637566	**2.6222**	**0.819841085**	0.51	0.499695247	**0.679**	**0.635689018**	**0.628147612**	**0.629048078**
pbmc68k	0.957231199	0.998446439	0.006770525	0.5367	**0.733781935**	0.5	0.494412826	**0.7945**	**0.711934669**	**0.709038396**	**0.704436566**
muris	**0.992922047**	**0.999185857**	**0.008719593**	0.6091	**0.731331262**	0.501566113	0.497313937	0.8123	0.747489462	0.738185475	0.738955564

cfDiffusion step10											
Dataset	SCC	PCC	Wasserstein	MMD	ILISI	KNN_AUC	KNN_ACC	RandomForest_AUC	RandomForest_ACC_Train	RandomForest_ACC_Test	RandomForest_OOB_Score
sapiens	**0.994067173**	0.998768687	**0.009065045**	**1.3768**	**0.781850093**	0.509784514	0.498641309	**0.8151**	**0.742603782**	**0.733697443**	**0.7342541**
Human_PF_Lung	**0.98804545**	0.997914191	**0.004915454**	**0.6067**	**0.561459654**	0.500633892	0.498258119	**0.7891**	**0.69556068**	**0.687516629**	**0.691650273**
WOT	**0.99276071**	0.999724106	**0.003526402**	2.6364	**0.815449907**	0.50578516	0.499695247	**0.6715**	**0.626410999**	**0.622021225**	**0.61970574**
pbmc68k	0.957269732	0.998455833	0.00674864	0.5399	**0.732473899**	0.5	0.494412826	0.7987	**0.710892637**	**0.711314985**	**0.70426667**
muris	**0.992990998**	**0.999202124**	**0.008142414**	0.6063	**0.759602832**	0.501663062	0.501566113	**0.7705**	**0.704703956**	**0.700092815**	**0.694378199**

cfDiffusion step20											
Dataset	SCC	PCC	Wasserstein	MMD	ILISI	KNN_AUC	KNN_ACC	RandomForest_AUC	RandomForest_ACC_Train	RandomForest_ACC_Test	RandomForest_OOB_Score
sapiens	**0.994075867**	0.99880135	**0.008237206**	**1.3959**	0.698414114	0.510212681	0.498872781	0.8307	0.757011693	**0.749140732**	**0.748646316**
Human_PF_Lung	**0.988076631**	0.997872327	**0.004761978**	0.6598	**0.561706787**	0.501213502	0.498258119	**0.7877**	**0.683934273**	**0.679897443**	**0.680056116**
WOT	**0.992806937**	0.99973409	**0.002969914**	**2.6452**	0.785613755	0.505804896	0.499715088	**0.664**	**0.620855443**	**0.613265798**	**0.614069786**
pbmc68k	0.957632417	0.998568799	**0.006461411**	0.5442	**0.725673938**	0.5	0.494412826	0.7988	**0.717269422**	**0.711552837**	**0.7104056**
muris	**0.992858114**	**0.999184823**	**0.007322305**	0.6026	**0.713122446**	0.502172513	0.4975184	**0.7454**	**0.691222683**	**0.682282671**	**0.681327512**

cfDiffusion step50											
Dataset	SCC	PCC	Wasserstein	MMD	ILISI	KNN_AUC	KNN_ACC	RandomForest_AUC	RandomForest_ACC_Train	RandomForest_ACC_Test	RandomForest_OOB_Score
sapiens	0.993958292	0.998698463	**0.006910331**	1.4232	0.279035266	0.511039026	0.499119682	0.8954	0.820638782	0.813220961	0.813419132
Human_PF_Lung	**0.987255996**	0.996954121	0.007064435	**0.3977**	**0.500786911**	0.512067791	0.499863386	0.8558	0.766318896	0.76092688	0.760554068
WOT	0.99296503	0.999726509	**0.002236074**	**2.589**	0.676548571	0.506002251	0.499715088	**0.738**	**0.673299566**	**0.665557164**	0.663016562
pbmc68k	**0.958673945**	0.99883236	**0.006129321**	**0.4143**	0.631348444	0.500024704	0.494412826	0.8109	0.729320753	0.727794767	0.720882556
muris	0.991469297	0.99876539	**0.008431928**	0.6333	0.322040547	0.510086803	0.499577268	0.8439	0.759133543	0.751007484	0.752476791

Supplementary Table 1. The data quality generated by cfDiffusion is influenced by the increment of jumping steps, exhibiting variation in its performance.

Supplementary Table 2. Average time cost for cfDiffusion to generate 6000 1000-dimensional latent features on muris, human_lung, and pbmc68k datasets, in seconds.

Jump Step	muris	human_lung	pbmc68k
5	166.79	169.01	163.77
10	159.50	162.44	157.34
20	151.26	157.72	154.17
50	147.23	153.57	148.08

Machine Configuration: Operating System: CentOS 7, Kernel Version: 3.10.0-1160.95.1.el7.x86_64; GPU Configuration: NVIDIA A100 80GB PCIe GPU, CUDA 11.2; Python Version: 3.8.

Supplementary Table 3. Comparison of cfDiffusion and scDiffusion simulation of muris_T_B data quality

Method	SCC↑	PCC↑	Wasserstein↓	MMD↓	ILISI↑
**cfDiffusion**	**0.98641**	**0.99908**	**0.01428**	**0.1029**	**0.69614**
scDiffusion	0.98541	0.99831	0.02019	0.1665	0.09313

Supplementary Table 4. Comparison of data quality of Homo Sapiens simulated by cfDiffusion and scDiffusion

Method	SCC↑	PCC↑	Wasserstein↓	MMD↓	ILISI↑
**cfDiffusion**	**0.99427**	**0.99903**	**0.00748**	2.6952	**0.83423**
scDiffusion	0.99390	0.99885	0.01092	**2.6807**	0.80365

Supplementary Table 5. Evaluating the realism of Homo Sapiens data simulated by two methods using random forest metrics

Method	AUC↓	ACC_Train↓	ACC_Test↓	OOB_Score↓
**cfDiffusion**	**0.81430**	**0.74514**	**0.73463**	**0.73605**
scDiffusion	0.84880	0.77570	0.76945	0.76718

Supplementary Table 6. Comparison of the quality of Alles data simulated by different methods

Method	SCC↑	PCC↑	Wasserstein↓	MMD↓	ILISI↑
**cfDiffusion**	**0.98629**	0.99811	0.031537783	**0.112**	**0.717895**

scDiffusion	0.986211	**0.99813**	**0.03143441**	0.114	0.672876
cscGAN	0.441771	0.91148	0.34375257	1.382	0
LSH-GAN	0.204212	0.42807	0.53537854	3.399	0
sciGAN	0.171575	0.02128	0.156193538	5.743	0

Supplementary Table 7. Comparison of the quality of Baron_Human data simulated by different methods

Method	SCC↑	PCC↑	Wasserstein↓	MMD↓	ILISI↑
**cfDiffusion**	**0.993203**	**0.99829**	**0.0223977**	**0.054**	**0.747422**
scDiffusion	0.99211	0.99816	0.023623938	0.062	0.702434
cscGAN	0.26003	0.46413	0.191790232	0.461	0
LSH-GAN	0.13095	0.21849	0.671651768	3.28	0
sciGAN	0.028022	-0.025	0.155322599	4.885	0

Supplementary Table 8. Comparison of the quality of Marshall data simulated by different methods

Method	SCC↑	PCC↑	Wasserstein↓	MMD↓	ILISI↑
**cfDiffusion**	**0.989488**	**0.9986**	**0.021281164**	**0.108**	**0.686555**
scDiffusion	0.98934	0.99858	0.021460413	0.109	0.554403
cscGAN	0.763284	0.92362	0.137774253	0.525	0
LSH-GAN	0.274762	0.39503	0.57576115	3.667	0
sciGAN	-0.016811	-0.0071	0.82472164	7.07	0

Supplementary Table 9. Comparison of the quality of Mizrak data simulated by different methods

Method	SCC↑	PCC↑	Wasserstein↓	MMD↓	ILISI↑
**cfDiffusion**	**0.977787**	**0.99798**	**0.009107215**	1.181	**0.71632**
scDiffusion	0.976378	0.99784	0.011517706	1.263	0.684844
cscGAN	-0.058571	0.32945	0.248307273	**0.554**	0
LSH-GAN	-0.163071	0.29282	0.282416199	2.261	0
sciGAN	0.154651	-0.0042	0.061417053	3.461	0

Supplementary Table 10. Assessment of the quality of WOT data simulated by two methods

Method	SCC↑	PCC↑	Wasserstein↓	MMD↓	ILISI↑	KNN_AUC↓	KNN_ACC↓
**cfDiffusion**	0.98569	0.9933	0.01637	**2.6222**	**0.81984**	0.51	0.49969
scDiffusion	**0.99261**	0.9997	**0.00457**	2.7052	0.79245	0.51	0.49969

Supplementary Table 11. Evaluation of the realism of WOT data simulated by two methods using random forests

Method	AUC↓	ACC_Train↓	ACC_Test↓	OOB_Score↓
**cfDiffusion**	**0.679**	**0.635689018**	**0.628147612**	**0.629048078**
scDiffusion	0.7444	0.679112397	0.685021708	0.67895964
