# Supplementary material for: cfDiffusion: diffusion-based efficient generation of high quality scRNA-seq data with classifier-free guidance
Source: Brief Bioinform. 2025 Feb 23;26(1):bbaf071. doi: 10.1093/bib/bbaf071 (PMC11846686; doi:10.1093/bib/bbaf071)
Supplement: Supplementary_Dataset_and_Model_bbaf071 [file supplementary_dataset_and_model_bbaf071.docx]

**Dataset Details**

Tabula Muris: RNA sequencing of single cells from 20 tissues of 3-month-old mice on the Illumina NovaSeq 6000 platform, from which single-cell data from 12 organs were selected, which included Bladder, Heart_and_Aorta, Kidney, Limb_Muscle, Liver,Lung, Mammary_Gland, Marrow, Spleen, Thymus, Tongue, Trachea. There are 57004 cells with 18996 genes per cell.

PBMC68k: peripheral blood mononuclear cells (PBMCs) are blood cells that are an important part of the immune system and are used to ward off infections and protect the body from harmful pathogens. In biomedical research, peripheral blood mononuclear cells are commonly used to study the global immune response to disease outbreaks and progression, pathogen infections, vaccine development, and a variety of other clinical applications. The dataset has a total of CD14+ Monocyte, CD19+B, CD34+, CD4+ T Helper2, CD4+/CD25 T Reg, CD4+/CD45RA+/CD25- Naïve T, CD4+/CD45RO+ Memory, CD56+ NK, CD8+ Cytotoxic T, CD8+/CD45RA+ Naïve Cytotoxic, Dendritic. The single-cell data for these 11 cell types totaled 68,579 cells with 17,789 genes per cell.

Waddington-OT: A cell reprogramming dataset of mouse embryonic fibroblasts (MEFs) was used to study the effects of induced pluripotent stem cells (iPSCs) and growth differentiation factor (GDF9) on reprogramming. The dataset contains cells with different timestamps during the 18-day reprogramming process. Data from three datasets were used and filtered out cells with expression counts of less than 10 and genes expressed in less than 3 cells, resulting in the retention of 82,920 cells with 19,423 genes per cell.

Homo Sapiens: Sequenced on Illumina NextSeq 500 platform, there are 6 types of cells, fibroblast, macrophage, memorybcell, naïvebcell, nkcell, plasmcell, they are from Bladder, Blood, Blood, Spleen, Thymus, Vasculature organs, the number of cells is 46177 and each cell has 28231 genes. Spleen, Thymus, Vasculature organs, the number of cells is 46,177 and each cell has 28,231 genes.

Human_PF_Lung: AT1, AT2, B Cells, Basal, Ciliated, Differentiating Ciliated, Endothelial Cells, Fibroblasts, HAS1 High Fibroblasts, KRT5-/KRT17+, Lymphatic Endothelial Cells, MUC5AC+ High, MUC5B+, Macrophages, Mast Cells, Mesothelial Cells, Monocytes, Myofibroblasts, NK Cells, PLIN2+ Fibroblasts, Plasma Cells, Proliferating Epithelial Cells, Proliferating Macrophages, Proliferating T Cells, SCGB3A2+, SCGB3A2+ SCGB1A1+, Smooth Muscle Cells, T Cells, Transitional AT2, cDCs, pDCs. These 31 cell types have a cell number of 114396 and 27281 genes per cell.

muris_mam_spl_T_B: consists of mammalian mammary T-cells, mammary B-cells, splenic T-cells, splenic B-cells, filtering out cells with expression counts of less than 10 and genes expressed in fewer than 3 cells, resulting in the retention of 11,330 cells with 14,652 genes per cell.

Alles: the Alles dataset consists of LVM longitudinal visc. muscle, amnioserosa, developing midgut, epidermis, fat body, germ cells, head mesoderm/hemocyte differentiation, midgut, muscle, neurogenesis, neurons, undifferentiated cells, visceral muscle, yolk. These 14 types of cellular compositions were filtered out with less than 10 expression counts and genes expressed in fewer than 3 cells, thus retaining 4614 genes. genes, thus retaining 4614 cells with 14850 genes per cell.

Baron Human: InDrop, a droplet-based single-cell RNA-seq method, was used to obtain the human pancreatic cell transcriptome with 14 types of cells: acinar, activated_stellate, alpha, beta, delta, ductal, endothelial, epsilon, gamma, macrophage, mast, quiescent_stellate, schwann, t_cell, filtered for expression counts. ductal, endothelial, epsilon, gamma, macrophage, mast, quiescent_stellate, schwann, t_cell, which are 14 cell types, and filtered out cells with expression counts of less than 10 and genes expressed in fewer than 3 cells, resulting in the retention of 8569 cells with 16359 genes per cell.

Marshall: Single-cell transcriptome data from Mus musculus' liver was sequenced on a 10X platform with a total of B1, B2, B3, B4, B5, B6, B7, CD8 T, Cd4 T, Monocyte, Monocyte derived Macrophage, NK, Red pulp Macrophage, Treg, cDC, pDC. These 16 cell types were filtered out to eliminate cells with expression counts of less than 10 and genes expressed in less than 3 cells, thus retaining 6022 cells with 16548 genes per cell.

Mizrak: profiled V-SVZ in 8-10 week old male and female mice using Drop-seq technique, a total of Astrocyte, Astrocytes, COP, Doublet, Endothelial, Ependymal, Microglia, Mural Cell + Fibroblast, Mural Cell+ Fibroblast, Mural+Fibroblast, Neuron, OPC, Oligodendrocyte, Oligodendrocytes, T Cell, aNSC+TAC+NB, T cell, which are 17 cell types totaling 42374 cells with 48529 genes per cell.

**Model Details**

The encoder of the AE model consists of three fully connected layers. The input data is processed through these layers to produce vectors of dimensions 1024, 1024, and 128, respectively. The decoder mirrors the encoder's structure, gradually reconstructing the 128-dimensional latent features back to the dimensions of 1024, 1024, and the original input data size. The diffusion backbone comprises nine fully connected layers, with dimensions sequentially set to 512, 512, 256, 128, 256, 512, 512, 1024, and finally 128. The decoder is structured symmetrically to the encoder and progressively reconstructs the 128-dimensional latent features back to the dimensions of 1024, 1024, and the original dimensions of the input data. Within the AE model, while the input and output dimensions vary according to the characteristics of different single-cell RNA sequencing (scRNA-seq) datasets, the other dimensions within the model remain fixed.
